# A Systematic Review of Techniques for Artifact Detection and Artifact Category Identification in Electroencephalography from Wearable Devices

**DOI:** 10.3390/s25185770

**Published:** 2025-09-16

**Authors:** Pasquale Arpaia, Matteo De Luca, Lucrezia Di Marino, Dunja Duran, Ludovica Gargiulo, Paola Lanteri, Nicola Moccaldi, Marco Nalin, Mauro Picciafuoco, Rachele Robbio, Elisa Visani

**Affiliations:** 1 Department of Electrical Engineering and Information Technology (DIETI), University of Naples Federico II, 80131 Naples, Italy; pasquale.arpaia@unina.it (P.A.); matteo.deluca@unina.it (M.D.L.); lucrezia.dimarino2@unina.it (L.D.M.); rachele.robbio@unina.it (R.R.); 2 Epileptology Unit, Magnetoencephalography Laboratory, Fondazione IRCCS Istituto Neurologico Carlo Besta, 20133 Milan, Italy; dunja.duran@istituto-besta.it (D.D.); elisa.visani@istituto-besta.it (E.V.); 3 Institute of Industrial Technologies and Automation, National Council of Research (STIIMA-CNR), 20133 Milan, Italy; ludovica.gargiulo@stiima.cnr.it; 4 Neurophysiology Unit, Fondazione IRCCS Istituto Neurologico Carlo Besta, 20133 Milan, Italy; paola.lanteri@istituto-besta.it; 5 ab medica S.p.A., 20023 Cerro Maggiore, Italy; nalin.marco@abmedica.it (M.N.); picciafuoco.mauro@abmedica.it (M.P.)

**Keywords:** EEG, artifact detection, artifact identification, artifact removal, wearable

## Abstract

Wearable electroencephalography (EEG) enables brain monitoring in real-world environments beyond clinical settings; however, the relaxed constraints of the acquisition setup often compromise signal quality. This review examines methods for artifact detection and for the identification of artifact categories (e.g., ocular) and specific sources (e.g., eye blink) in wearable EEG. A systematic search was conducted across six databases using the query: (“electroencephalographic” OR “electroencephalography” OR “EEG”) AND (“Artifact detection” OR “Artifact identification” OR “Artifact removal” OR “Artifact rejection”) AND “wearable”. Following PRISMA guidelines, 58 studies were included. Artifacts in wearable EEG exhibit specific features due to dry electrodes, reduced scalp coverage, and subject mobility, yet only a few studies explicitly address these peculiarities. Most pipelines integrate detection and removal phases but rarely separate their impact on performance metrics, mainly accuracy (71%) when the clean signal is the reference and selectivity (63%), assessed with respect to physiological signal. Wavelet transforms and ICA, often using thresholding as a decision rule, are among the most frequently used techniques for managing ocular and muscular artifacts. ASR-based pipelines are widely applied for ocular, movement, and instrumental artifacts. Deep learning approaches are emerging, especially for muscular and motion artifacts, with promising applications in real-time settings. Auxiliary sensors (e.g., IMUs) are still underutilized despite their potential in enhancing artifact detection under ecological conditions. Only two studies addressed artifact category identification. A mapping of validated pipelines per artifact type and a survey of public datasets are provided to support benchmarking and reproducibility.

## 1. Introduction

The application of EEG has evolved progressively over time, expanding across distinct domains [1]. Initially, EEG was predominantly employed in clinical settings [2,3], especially for the diagnosis of neurological disorders such as epilepsy [4], Alzheimer’s disease [5], Parkinson’s disease [6], brain tumors [7,8,9], and stroke [10]. In parallel, EEG was widely used in neuroscience research to investigate nervous system functionality and brain dynamics, as well as to identify correlations between neural rhythms and specific cognitive or sensory states [11,12,13]. In subsequent years, EEG gained traction in psychological contexts, particularly through the development of neurofeedback interventions. In these applications, real-time visual or auditory feedback of neural activity is used to promote self-regulation skills [14,15]. Neurofeedback has been applied to support the treatment of attention and learning disorders, substance use disorders, traumatic brain injuries, and Autism Spectrum Disorder (ASD) [15,16] as well as to enhance cognitive and motor performance in healthy individuals such as surgeons [17] and athletes [18]. In recent decades, EEG-based Brain–Computer Interfaces (BCIs) have been developed, including the P300 speller [19] and motor-imagery-based systems [20], with the aim of facilitating communication and control of external devices.

Nowadays, in addition to well-established domains such as (i) neuroscience research and (ii) healthcare, EEG is increasingly being applied across a wide range of emerging fields, including (iii) well-being and mental health [21,22,23], (iv) entertainment, such as the development of medical-oriented games, attention-monitoring systems, and interfaces for controlling drones or humanoid robots [24,25,26], (v) industrial settings, where EEG-based BCIs support safety, efficiency, and decision-making by tracking cognitive states, e.g., fatigue, stress, or alertness, particularly in repetitive or high-risk tasks using collaborative robots and machines [21,27], and (vi) professional and competitive sports, for the assessment and enhancement of motor performance [28,29]. The expansion of EEG applications into a broader range of non-clinical domains has been made possible by the development of portable and wearable systems [22,30,31,32]. This trend has driven a strong acceleration in the wearable BCI market, with projected compound annual growth rates ranging from 8% to 17% over the next decade [33,34].

In the healthcare sector, the emergence of the *community medicine* paradigm has fostered interest in accessible technologies offering diagnosis and therapy at the patient’s home. In this context, wearable EEG devices are regarded as a promising solution for expanding access to medical interventions in rehabilitation, as well as for enabling early and cost-effective screening across large segments of the population, thus representing a significant opportunity for public health [35,36,37,38]. In addition, within the context of personalized medicine, wearable EEG devices enable real-time monitoring of therapeutic protocols, allowing dynamic adjustments to enhance treatment effectiveness [39,40]. Furthermore, the ecological relevance of the measurement based on wearable technologies is improved, and psychologists consider EEG as a promising new psychometric tool for capturing cognitive and emotional states in real-world environments [41,42].

Although wearable EEG offers significant opportunities, caution persists within the clinical and neuroscientific communities, primarily because of concerns regarding its lower signal quality relative to conventional EEG systems [43,44]. The main factors contributing to signal degradation in wearable EEG systems are (i) uncontrolled environments, (ii) in-motion conditions, and (iii) the adoption of dry or semi-wet electrodes for rapid setup. Operation in everyday environments limits the experimenter’s ability to mitigate environmental noise, such as electromagnetic interference [45], exposes the system to the effects of natural movements, including high-intensity motion permitted to the user, and results in reduced electrode stability due to the absence of conductive gel [46]. Additionally, the reduced number of channels, typically below sixteen [47], limits spatial resolution and impairs the effectiveness of standard artifact rejection techniques based on source separation methods, such as Principal Component Analysis (PCA) and Independent Component Analysis (ICA) [48].

Most existing reviews on artifact detection target high-density EEG and physiological sources, in particular ocular, muscular, and cardiac [49,50,51,52]. Only a few surveys extend this analysis to non-physiological artifacts [53,54,55]. Limited attention has been dedicated to evaluating algorithms designed for the specific artifact properties of wearable EEG systems. A notable exception is the study by Seok et al. (2021) [56], presenting a structured overview of techniques for wearable EEG and photoplethysmography signal processing; however, its scope remains restricted to motion-related artifacts. The present review provides a systematic survey of methods designed to detect artifacts and to identify the specific artifact categories within EEG signals acquired through wearable devices. With respect to the previous literature, this review expands the scope by including a wider range of artifacts observed in wearable devices rather than focusing only on specific types. Moreover, category artifact identification assumes a novel relevance. Accurate identification of artifact categories is a critical step in enhancing signal quality in wearable EEG systems. Each artifact type exhibits distinct spatial, temporal, and spectral characteristics that require tailored detection and removal strategies [54,55,57]. Without clear classification, processing pipelines risk applying overly generic solutions, which can be ineffective and may even compromise the integrity of neurophysiological components of interest [58,59]. Artifact categorization also enables the design of modular and adaptive pipelines capable of addressing the complex and dynamic conditions typical of real-world recordings, including low-density or single-channel configurations [60,61]. Such advancements are crucial to improving the reliability of wearable EEG analyses in both clinical and applied settings.

This review specifically focuses on wearable EEG devices and addresses the following research questions (RQs):

- RQ-I: have any studies addressed the specific challenges of artifact management in wearable EEG systems?
- RQ-II: which algorithms are available in the literature for artifact detection and artifact category identification in EEG signals acquired by wearable devices?
- RQ-III: which parameters are used to assess the performance of artifact detection and artifact category identification algorithms?
- RQ-IV: which assessment metrics are employed, and which reference signals are used to assess the performance of artifact detection and artifact category identification algorithms?

## 2. Materials and Methods

### Search Strategy

The literature search was conducted across the databases Google Scholar, Scopus, PubMed, IEEE Xplore, Science Direct, and Web of Science by applying the following combined query in all searchable fields: (“electroencephalographic” OR “electroencephalography” OR “EEG”) AND (“Artifact detection” OR “Artifact identification” OR “Artifact removal” OR “Artifact rejection”) AND “wearable”. No restrictions were imposed on the year of publication. The most recent search was conducted on 24 April 2025. The article selection process was carried out systematically and transparently, following the PRISMA guidelines [62]. During the pre-screening phase, only peer-reviewed articles published in journals or conference proceedings, written in English, and with full-text availability were considered, then the duplicates were excluded. In the screening phase, titles and abstracts were reviewed to eliminate studies considered irrelevant or inconsistent with the search query. Finally, during the eligibility phase, the full texts of the remaining studies were assessed based on the following exclusion criteria: (i) studies not focusing on wearable EEG systems (defined by having sixteen or fewer channels, or using dry electrodes, or assessing performance considering hardware efficiency metrics (power consumption, silicon area, and computational burden)); (ii) studies not referring to EEG artifact management; (iii) animal studies. An additional screening was conducted on the reference lists of the screened studies to further enhance the sample size and ensure comprehensive coverage of the literature. Only articles meeting all criteria were included in the final review to address the research questions. Data extracted from the included studies were organized using a standardized Excel template specifically designed by the research team. Four reviewers independently charted the data. The template was initially pilot-tested on a subset of four studies (i.e., one study per reviewer) to verify clarity and ensure consistency in data collection. Discrepancies were resolved through discussion and consensus, with the involvement of a third reviewer when consensus could not be reached. The PRISMA checklist is provided in the Supplementary Materials to ensure transparency and adherence to reporting guidelines.

## 3. Results

The articles selection procedure is presented in Section 3.1. The temporal publication trend of the studies is discussed in Section 3.2, where it is compared with publication trend in generic wearable EEG device studies. Section 3.3 offers a detailed overview about the acquisition setup and performance assessment methods of the algorithms proposed by the selected studies.The Section 3.4 maps the artifact detection pipelines proposed in the literature for wearable EEG. Moreover, Section 3.5 defines the artifact detection and artifact category identification strategies among the studies.

### 3.1. PRISMA

**Figure 1:**
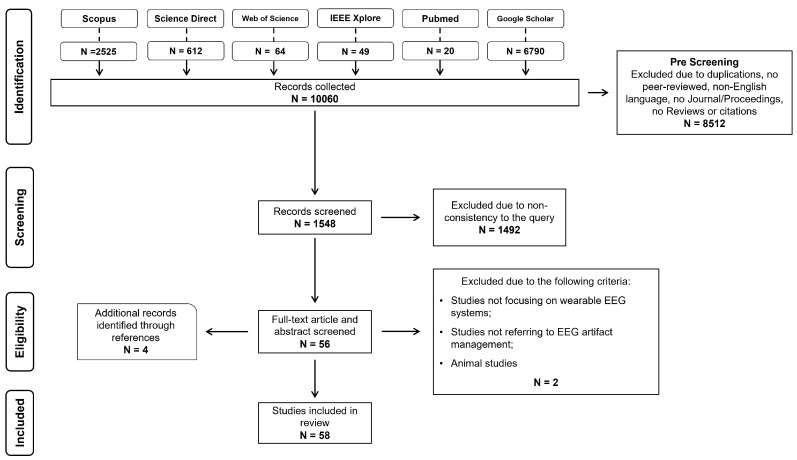
PRISMA—flow of articles selection process.

A flow diagram detailing the results of identification, screening, eligibility, and inclusion phases is provided in Figure 1. The query applied to the six databases allowed the identification of 10,060 articles. After duplicate removal and the application of pre-screening criteria, 8512 records were excluded. The remaining 1548 articles underwent title- and abstract-based screening, leading to the exclusion of an additional 1492 studies. During the eligibility phase, full-text evaluation was conducted on 56 potentially relevant articles, with 2 further exclusions based on predefined exclusion criteria. To ensure comprehensive coverage, reference lists of the included studies were reviewed, resulting in the addition of four articles not captured in the initial search. In total, 58 studies met all criteria and were included in the qualitative analysis. To overcome the limited number of recent studies employing deep learning approaches identified in the initial search (only 2 papers after 2023), a modified search strategy was implemented [63]. The keyword ‘wearable’ was removed to relax the inclusion criteria and the keyword ‘deep learning’ was added with the operator ‘AND’ to increase the likelihood of identifying new and innovative deep learning solutions published after 2023. Consistency with the scope of the review was preserved by ensuring that all devices in the retrieved studies could be qualified as wearable systems, defined as employing fewer than sixteen channels or using dry electrodes. This modified search resulted in the inclusion of four additional studies.

### 3.2. Temporal Trends in Wearable EEG and Artifact Management

**Figure 2:**
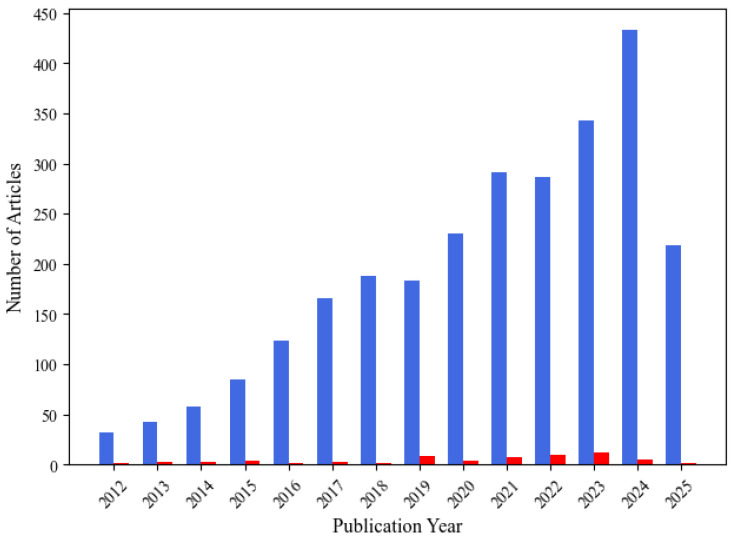
Comparison of publication trends between articles on wearable EEG (blue) extracted from Scopus and studies collected by this review focusing on artifact detection and removal in wearable EEG (red). Artifact-related issues in wearable EEG remain underexplored, with a stagnating publication trend.

The analysis of the publication year for the collected articles reveals an almost stagnant publication trend. As an illustrative case, an advanced search on Scopus using the query (‘EEG’ AND ‘wearable’) was applied to titles, keywords, and abstracts. The analysis prioritized databases yielding a higher number of results. Among them, Scopus was selected over Google Scholar for further investigation, as it allows for field-specific searches and provides results with greater relevance to the research query. Figure 2 shows a comparison of publication trends between articles on wearable EEG (blue) extracted from Scopus and studies collected in this review focusing on artifact detection and removal in wearable EEG (red).

The overall number of publications related to wearable EEG devices has steadily increased over the last decade, reaching approximately 440 articles in 2024. This trend confirms the expanding interest in wearable EEG across clinical, research, and applied contexts while simultaneously highlighting the limited attention devoted to artifact management. The observed discrepancy points to a significant gap in the current literature: the growing use of wearable EEG contrasts with the limited focus on artifact contamination, hindering the development of effective removal methods and the acquisition of reliable, high-quality signals.

### 3.3. Acquisition Setup and Performance Assessment Methods of the Algorithms Across the Studies

The current Section focuses on the main contents provided by the reviewed articles. In particular, Section 3.3.1 reports the description of the grid adopted to extract specific parameters from articles. Section 3.3.2 presents the results related to the acquisition setup parameters, whereas Section 3.3.3 details the results concerning the methods used to assess the algorithm performance.

#### 3.3.1. Grid for Collecting Acquisition Setup Parameters and Performance Assessment Methods

The configuration of acquisition systems and methods for algorithms performance assessment, reported in the included studies, are provided in Table 1. For each study, the targeted artifacts are specified, including their category and specific source, to outline the application domain of the algorithms. Experimental data are categorized as real (R) recordings, fully simulated (S) signals, or semi-simulated (SS) signals, the latter obtained by combining simulated or real artifacts with clean real recordings. For R and SS data, the table reports the number of subjects, number and duration of trials, and the specific task or experimental condition. For S data, only the trial structure is provided, as subject-specific details are not applicable. Channel configurations, including the number, type, and electrode placement, are detailed for R and SS data, while for S signals, only the number of channels processed by the algorithm is indicated. Subsequent columns report the reference signal used for validation, alongside performance parameters and metrics. The assessment parameters and corresponding metrics reported in the studies refer to the overall performance of the algorithms, generally including both artifact detection and removal. Consequently, although the metrics are directly associated with the output of the removal phase, they can also provide an indirect assessment of the detection phase, as the latter represents an essential preliminary step for the removal process. The final column *Algorithm* retains the original nomenclature used in each study for the proposed methods, typically including both detection and removal stages.

#### 3.3.2. Results of the Acquisition Setup Parameter Collection

The artifacts examined in the studies are reported according to their categories and sources (see *Focused Artifact* in Table 1). The artifact categories include ocular, muscular, cardiac, instrumental, Electromagnetic Interference (EMI), movement, and an unspecified category labeled as *other*.

Concerning the sources, for ocular artifacts, a distinction is made between eye blinks and eye movements. Eye blinks are transient events caused by rapid eyelid closure, generating large, short-lived deflections in the EEG signal [127]. In contrast, eye movements involve voluntary or involuntary changes in gaze direction, characterized by longer durations and lower frequencies [127,128]. Muscular artifacts originate from Muscle Contraction (MC) contaminating the EEG signal, often associated with specific actions, usually involving multiple muscle groups simultaneously. For example, jaw motion engages the orbicularis oris, masseter, and temporalis, while chewing primarily activates the masseter and temporalis. Accordingly, sources of muscular artifacts include MC of Masseter (Ma), Temporalis (Te), Corrugator Supercilii (CS), Zygomaticus (Z), Orbicularis Oris (OOr), Orbicularis Oculi (OOc), Limb (Li), Nasalis (N), Submentalis (Subm), Shoulder (Sh), Pharyngeal (P), and Tongue (To). EMI artifacts mainly refer to Power-Line Noise (PLN) (50 or 60 Hz, depending on the geographical location of the recording source) [127,129]. Instrumental artifacts sources are (i) electrode pop, due to sudden changes in impedance at the electrode–scalp interface or unstable contacts [127,129]; (ii) electrode displacement, caused by the electrode moving relative to the scalp [127]; (iii) Electrode Impedance Imbalance (EII), linked to variations in impedance or potential at the electrode–skin interface, frequently due to poor contacts, damaged cables, or insufficient use of conductive gel [127,128]; (iv) Thermal Electronics Noise (TEN), generated by the resistance of electronic components, characterized by a flat frequency spectrum [127]; (v) clipping, occurring when the output voltage exceeds the amplifier’s handling capacity, resulting in a truncated signal [130]. Instead, movement artifact sources correspond to (i) body movements [129,131] (e.g., during walking or running); (ii) head movements [128] (e.g., head shaking); (iii) limb movements during daily activities [127,129] (e.g., typing or grasping objects); (iv) tremor [128], either physiological or pathological (e.g., Parkinson’s disease [131]). In cases where only the artifact category is provided, the related source information is denoted as *Source Not Specified* (*S.N.S.*).

**Figure 3:**
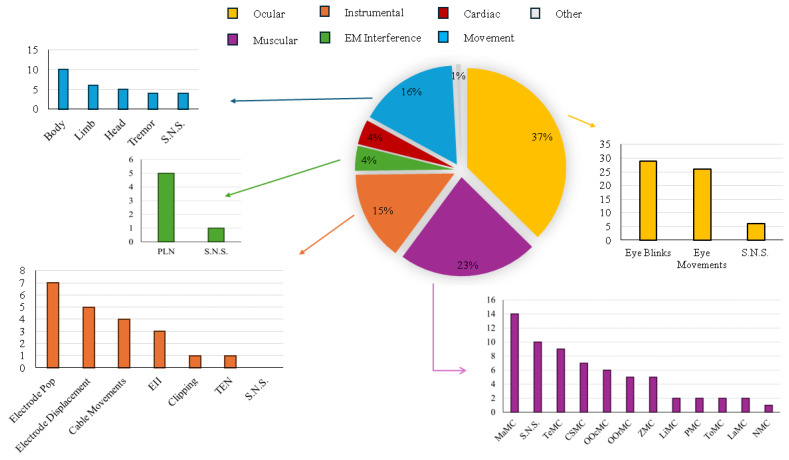
Pie chart of the percentage distribution of artifact categories addressed in the reviewed articles. For each category, a corresponding bar chart indicates its corresponding sources. “Source Not Specified” (S.N.S.) is used when the artifact category is indicated without explicit information on its source. An explanatory table for other technical acronyms is recommended at the end of the document.

In particular, Figure 3 illustrates the percentage distribution of artifact categories and the corresponding sources explored. Ocular artifact represents the most frequently studied category (37%), confirming the priority given to removing eye blinks and eye movements, addressed in 29 and 26 studies, respectively. Muscular artifacts represent the second most addressed category (23%), although in many cases (10) the specific muscle location remains unreported. When specified, the most frequent sources include MaMC (14 cases), TeMC (9 cases), and CSMC (7 cases), followed by ZMC (5 cases), OOrMC (5 cases), and OOcMC (5 cases), with isolated cases for LiMC, NMC, SubMC, ShMC, PMC, and ToMC. Movement artifacts represent 16% of cases (10 studies evaluated body movements, 5 studies head movements, 6 studies limb movements, and 4 studies tremor). Instrumental artifacts represent 15% of cases, primarily related to cable movements (4 studies), electrode dislocations (5 studies), electrical impedance imbalances (3 studies), and electronic thermal noise (1 study). Instead, EMI, specifically PLN, represents 4% of cases. Finally, cardiac artifacts account for approximately 4% of cases (6 studies). The *other* category appears in a single study, Casadei et al. (2020) [89], analyzing artifacts characterized by large discontinuities in alpha activity. This study proposes a preliminary method applied to clinical EEG signals, highlighting its potential for long-term monitoring with wearable EEG, where such artifacts are more frequent. Additionally, the label *S.N.S.* appears in approximately 20 cases, most frequently for muscular artifacts, affecting the reproducibility of the proposed solution.

Regarding sample size (see *Experimental Sample* in Table 1), the studies range from single-subject designs [89] to cohorts with over 100 participants [60,74,99,102,104,121,125,126], while the majority (66%) include between 5 and 30 subjects. Approximately 61% of the recordings are collected in resting-state or sleeping conditions, while the remaining 39% involve cognitive and/or motor task executions. In terms of data type, 59% of the studies employ R, 11% use fully S, and 30% rely on SS. Some studies validate the proposed algorithm on several datasets of the same type, as in Grosselin et al. (2019) [83] and Kumaravel et al. (2023) [115]. Others assess performance across different data types, testing the same algorithm on both R and S signals [66,68,71,76,95,108,109] or on R and SS data [90,100,116,132]. As regards the number of electrodes, in R and SS datasets, four studies use single-channel devices [65,89,101,113] and seven focus on a single channel extracted from multi-channel recordings [60,61,96,104,109,116,120]. Three additional studies apply their methods to a limited number of channels compatible with wearable systems [85,107,115]. A total of 36 studies employ acquisition systems with 2 to 16 channels. Some studies include devices with more than 16 electrodes (19 [92,122], 21 [90], and 22 [106]), taken into account in the analysis due to their explicit focus on wearable EEG, either by using dry electrodes [53] or by addressing hardware efficiency metrics such as power consumption, silicon area, and computational load [92,106,122]. Finally, in seven studies, the number of electrodes is not specified. Concerning electrode type, wet configurations remain predominant, covering approximately 61% of the sample, while the use of dry sensors reaches 24%. In the remaining 15% of cases, details about the electrode type are not reported. In S datasets, no physical acquisition setup can be involved, with all reviewed studies processing a single virtual channel in parallel.

#### 3.3.3. Results of the Performance Assessment Methods Collection

Methods performance is assessed using a reference signal, specific parameters, and quantitative metrics. Some studies rely on self-produced data, while others make use of publicly available datasets reported in Table 2.

The public datasets are categorized by data type, signal category, experimental protocol, participants, hardware setup, and signal processing phase. Despite the availability of most public datasets, a few were not accessible. Specifically, the EEGlab dataset [143] could not be retrieved, while the dataset by [142] is available only upon request. Among all the datasets examined, only one was explicitly developed to model movement-related artifacts typical of wearable devices [64]. The majority of the public datasets include R data, whereas only one [114] also provides SS data. In terms of signal category, there is a clear predominance of physiological EEG data, often recorded during cognitive or motor tasks, or clinical EEG data, containing recordings from patients with neurological conditions (e.g., epilepsy or stroke). Several datasets also provide physiological (Electrooculogram (EOG), Electromyogram (EMG), Electrocardiogram (ECG)) or instrumental (cable movement) artifacts. In some cases, the artifact signals are recorded in a controlled manner, using protocols designed to induce eye blinks, eye movements, or cable movement. Whether using self-produced or public data, three main categories of reference signals can be identified across studies: (i) clean signal (S or R), (ii) artifact (S or R), and (iii) physiological signal (R). The clean signal can be simulated or real. In the case of simulated clean signals, Peng et al. (2013) [66] and Zhao et al. (2014) [68] refer to the simulation method proposed by [144], who generated clean EEG signals containing 1280 samples (5 s duration, 256 Hz sampling frequency) by filtering white noise through a seventh-order autoregressive model, established based on segments of real EEG recordings not containing visible artifacts. Rahman et al. (2015) [71] adopted a simulation approach originally introduced by Yeung et al. (2004) [145], although with a different purpose. In the former, the simulated signal was used as a reference to assess the performance of the eye-blink artifact removal method proposed, whereas in the latter, the obtained signal was employed to assess the validity of analytical methods previously employed to demonstrate that event-related potential (ERP) peaks originate from the synchronization of ongoing EEG oscillations. In both cases, EEG epochs (from −400 to 400 ms, 250 Hz sampling rate) were simulated by adding two phasic ERP peaks to background EEG noise constructed by summing 50 sinusoids with random frequencies (0.1–125 Hz) and phases (0–2π) and a maximum amplitude of 20 μV at 0.1 Hz. In both cases, the clean signal serves as reference for artifact removal algorithm performance assessment, mainly in terms of accuracy. In the case of real clean signal, reference selection occurs either before (ex ante) or after (ex post) signal acquisition, enabling performance assessment in terms of accuracy on both R and SS data. In ex ante cases, 30 or 60 s segments recorded before artifact onset from the same participant are used. During the acquisition, the subject remains still and relaxed to reduce movement-related artifacts [67,68,78,85,90]. In other cases, to reduce ocular artifacts, reference data are commonly acquired during cross fixation tasks [107,123] or under resting-state conditions with eyes open [113] or closed [96]. An alternative reference is obtained involving controlled experimental settings: Sweeney et al. (2012) [64] and Mahmud et al. (2023) [112] used an uncontaminated channel as reference by voluntarily generating the artifact on one channel (experimenter moves the target channel cable) and retaining the other for comparison. In ex post cases, clean signal segments are selected after acquisition. Some studies rely on visual inspection of the signal [88,100], occasionally supported by synchronized video [59]. In several cases, clean signal is obtained by using traditional artifact removal methods, particularly ICA, eventually combined with automated classification tools such as ICLabel [60,100,102,111,116,125,132]. In one case [94], root mean square (RMS)-based criteria are applied to identify artifact-free segments. Similarly, the artifact signal can also be either simulated or real. Simulated artifacts are presented in only one study [76], aimed at evaluating the algorithm’s capability to remove artifacts by comparing original and residual artifact amplitudes. In this case, the artifact is simulated using the Markov Process Amplitude (MPA) EEG model, where EEG oscillations (delta, theta, alpha, beta) are modeled as sinusoids with amplitudes governed by a first-order Gaussian–Markov stochastic process, parameterized to replicate the power spectrum of real EEG. In the case of real artifacts, contaminated signals are mainly used to evaluate classification metrics in order to assess detection performance. In Jayas et al. (2023) [110], artifact annotation is automatically performed using built-in functions of the MNE-Python package, which applies predefined thresholds to detect EOG and EMG signals. In other studies [68,113], labels are manually assigned after artificially adding artifacts to previously clean signals. In Peh et al. (2022) [104] and Zhang et al. (2022) [109], pre-labeled datasets are used. In D’Rozario et al. (2015) [72], labeling is conducted through visual inspection of the EEG signal. The physiological signal, generally labeled as raw data in the articles, is used as a reference for the assessment of removal algorithm selectivity, assuming a signal not completely artifact-free. In most cases, the reference is obtained through controlled experimental protocols, where artifact generation is guided while leaving portions of physiological signal intact. Majmudar et al. (2015) [69] include an initial resting-state phase with eyes closed (10 s), followed by a phase involving blinking every 5 s. Chen et al. (2022) [100] provide vocal instructions to induce specific artifacts (blinking, chewing, frowning, eye/head movements) during six runs, each separated by 60 s of rest. Occhipinti et al. (2022) [101] define two distinct recording stages: a 4 min resting phase (2 with eyes open and 2 with eyes closed) and an active phase including artifact-inducing activities (speaking, chewing, walking in place at 120 bpm), each lasting 2 min. On the other hand, in some cases [58,65,66,84,108,116], no instructions during acquisition are explicitly described. Additionally, in studies incorporating auxiliary sensors, EOG [71,73] or EMG [93] channels serve as references.

In terms of assessment parameters, accuracy (defined as the distance between the measured value and the true value) emerges as the most commonly used, being reported in 71% of the studies. In particular, accuracy is related to metrics such as RMS, Root Mean Squared Error (RMSE), Relative Root Mean Squared Error (RRMSE), Mean Square Error (MSE), or Mean Absolute Error (MAE) values (47% of the studies), followed by power or Signal-to-Noise Ratios (SNRs), including Difference in Signal-to-Noise Ratio (DSNR) and Peak Signal-to-Noise Ratio (PSNR), present in 29% of studies. Metrics with less than 10% frequency include Dynamic Time Warping (DTW), artifact reduction rate, spectral indices (Spectral Score, Distribution Score, Power Spectral Density (PSD)-based, Amplitude Modulation Rate of Change (AMRC), accuracy gain, amplitude or phase coherence, Zero Crossing Rate (ZCR), and Max-gradient). On the other hand, the parameter selectivity is reported in 63% of studies, based 45% on correlation metrics (Pearson’s correlation, Correlation Coefficient (CC), or coherence), followed by power or spectral metrics (23% of the studies), Mutual Information (MI) (10% of the studies), and Magnitude Square Coherence (MSC) plot (3% of the studies). Finally, distribution statistics such as skewness, kurtosis, or Autocorrelation Function (ACF) appear in 5% of the studies. Instead, classification performance metrics are evaluated in 29% of the studies. The most commonly reported metrics include classification accuracy (19% of the studies), True Positive Rate (TPR) (15% of the studies), and False Positive Rate (FPR) (12% of the studies). Precision and recall are reported in 3% of the studies, while False Positives per Minute (FPM) and False Positives per Hour in 3% of the studies. Metrics, such as Cohen’s k and Positive Predictive Value (PPV), are each employed in only one study, conducted by D’Rozario et al. (2015) [72] and Zhang et al. (2022) [109], respectively. Another parameter is operational speed, based on latency as metrics, assessed in 13% of the studies. Moreover, the hardware efficiency parameter appears in 7% of studies. In particular, the related metric power consumption occurs in 4% of the studies, while the metrics computational burden and silicon area are each considered in only one study, conducted by Xing et al. (2024) [132] and Bahadur et al. (2024) [122], respectively. Only one study [74] includes a clinical efficacy parameter, represented by a self-rating score for depression risk.

Finally, the algorithms (including both artifact detection and removal stages) are reported according to the classification proposed in [57]. Specifically, the classification relies on the main method adopted, disregarding the decision rule method, also in order to achieve a more synthetic classification. The classes of methods considered in the reviewed studies are filtering methods, blind source separation methods, source decomposition methods, and combinations of different algorithms. In particular, filtering methods consist of conventional or adaptive filters, including Kalman filter, Adaptive Noise Canceler (ANC), Least Mean Square (LMS), Recursive Least-Square (RLS), or multi-channel adaptive filter (MCAF). Blind source separation methods refer to techniques employing ICA, Canonical Correlation Analysis (CCA), and Singular Spectrum Analysis (SSA). Instead, source decomposition methods include algorithms based on wavelet transform, Empirical Mode Decomposition (EMD), Ensemble Empirical Mode Decomposition (EEMD), Multivariate Empirical Mode Decomposition (MEMD), Fast Multivariate Empirical Mode Decomposition (FMEMD), Variational Mode Extraction (VME), or Morphological Component Analysis (MCA). Finally, the combinations of different algorithms may involve Wavelet-ICA (wICA), Discrete Wavelet Transform (DWT), and ANC or methods involving machine learning algorithms (e.g., Support Vector Machine (SVM), K-means, decision trees, random and gradient boosting) based on supervised/unsupervised learning applied to temporal or frequency-domain features. In addition to the aforementioned classes, a group of approaches not addressed in these classifications can be identified, particularly, deep learning methods, which include a variety of neural network models for artifact detection and removal (e.g., Convolutional Neural Network (CNN), Gated Recurrent Unit (GRU), Multilayer Perceptron (MLP), autoencoders, Generative Adversarial Network (GAN), EEGIFNet); ASR-based methods (e.g., ASR, rASR, MEMD-ASR). Furthermore, several studies propose individual algorithms that do not fit neatly into the proposed classes, using specific mathematical or statistical strategies. Examples include a model-based amplitude estimation [89] extracting components of a modeled alpha sinusoidal wave, an algorithm based on statistical features for artifact detection [72] identifying segments using predefined thresholds on standard deviation, a strategy by evaluating several morphological parameters (e.g., amplitude, symmetry, slope) [59], a Multiscale modified-distribution entropy (M-mDistEn) algorithm [97] based on entropy measure quantifying signal irregularity across multiple time scales, an approach combining features such as trend slope, standard deviation, peaky distribution, and spectral power to detect EEG segments with artifacts (using *pop_rejtrend, pop_jointprob, pop_rejkurt, pop_rejcont, EEGLAB* functions) [75].

**Figure 4:**
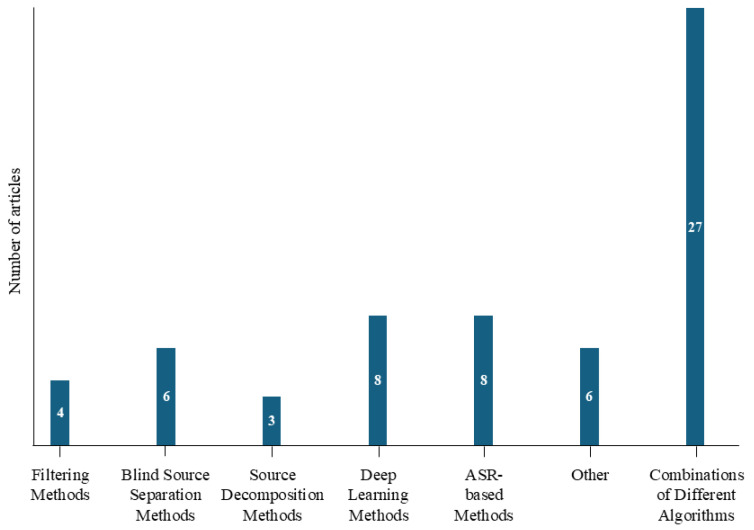
Number of studies addressing a specific algorithm class, according to the classification proposed in [57]. The classes *Deep Learning methods*, *ASR-based methods* and *Other* are included to account for algorithms that can not be mapped to the classes in [57] (e.g., [59,72,75,89,97]).

Figure 4 illustrates the distribution of algorithm classes across the reviewed studies, with combinations of different algorithms emerging as the most prevalent, and source decomposition methods being the least employed.

Focusing on the performance results, large variability emerges across studies due to differences in artifact considered, recording setup, and algorithmic strategy. Filtering and wavelet-based methods typically report SNR improvements between 5 and 15 dB for ocular artifacts, while others (e.g., Kim et al. (2015) [70] using ANC on motor-task data) observed marginal or even negative improvements depending on task complexity. RMSE and RRMSE values usually fall within 0.1–0.6. Lower errors are reported for wavelet–thresholding on ocular artifacts (Shahbakhti et al. (2021) [95], Zhang et al. (2022) [109]), whereas higher values are observed in semi-simulated datasets containing several or muscular artifacts (e.g., Cheng et al. (2019) [78], Butkevičiūtė et al. (2019) [86], Islam et al. (2020) [90]). Classification-based performance reach accuracies above 90% in several cases (e.g., D’Rozario et al. (2015) [72], Ingolfsson et al. (2024) [124], Chen et al. (2022) [100]). Latency values, when provided, range from tens of milliseconds in lightweight filtering (e.g., Matiko et al. (2013) [65]) with Adaptive Predictive Filtering (APF) + (DWT) to several seconds in more complex frameworks such as ASR or Deep Learning-based methods (Blum et al. (2019) [85], Zhang et al. (2022) [109]).

### 3.4. Parameters of Artifact Detection Pipelines Across the Studies

In this Section, a focus on the detection algorithms is made. In particular, the parameters used at each stage of the detection pipeline are presented (Table 3). The table outlines the pre-processing, the epoching, the feature extraction, the feature selection, and the classification methods or decision rules used to differentiate artifact-contaminated segments from clean data or identify a specific artifact source. Finally, it reports the spatial specificity of the detection process in terms of the channels involved: (i) single-channel, operating on individual channels independently; (ii) multi-channels, using information from several channels without processing the entire montage; or (iii) all-channels, simultaneously analyzing all available channels. A focus on ASR algorithm, considered relevant for wearable EEG devices, is presented in Section 3.4.1.

Pre-processing procedures across studies commonly involve the application of band-pass, high-pass, and low-pass filters. Band-pass filters are frequently set within 0.5–40 Hz to preserve EEG bands of interest and reduce drift and power line noise [66,68,122], with upper limits extending to 60–64 Hz [59,90] and in some workflows up to 70 Hz [78,88,102,113]. High-pass filters are commonly configured with cut-off frequencies at 0.1 Hz [73], 0.25 Hz [85], 0.3 Hz [72], 0.5 Hz [96,100,110], or 1 Hz [75,94,104,123,132]. Low-pass filters are generally applied with cut-off values between 35 and 45 Hz [72,85,94,115], although some studies extend this range up to 60–64 Hz for the analysis of lower gamma band activity [90]. A notch filter at 50 or 60 Hz is also commonly employed (17 studies) to suppress power line interference. In some cases, it is applied alongside a band-pass filter with an upper cut-off below 50 Hz, aiming to eliminate residual noise persisting after preliminary filtering [72,101,116]. In other cases, a broader band-pass filter extending up to 65 Hz is used, with the notch filter selectively removing the power line component within this frequency range [53,60,82,102].

Additional steps, including normalization and detrending, are incorporated in certain pipelines to optimize signals for feature extraction or neural network input [125,132]. Pre-processing is sometimes applied uniformly across signals, either clean or with artifact [66,90,113], while in other workflows, each type of signal is pre-processed differently [60,78,83,88,93,100,102,132].

In terms of epoching, approximately 56% of studies employ windows with durations of 2 s or less. Excluding the 20% of cases without reported details, the remaining workflows typically use windows up to 10 s, with the exception of Peng et al. (2013) [66] and Dey et al. (2020) [92], analyzing epochs of 40 and 128 s, respectively. Several studies [59,84,89,105,110,112,119,124] investigate the influence of window length on algorithm performance by testing multiple lengths. Instead, Kumaravel et al. (2021) [94] examine the role of overlapping in artifact detection, using 10 s windows with overlap percentages ranging from 50% to 75%.

With regard to feature extraction and classification, one of the most frequent approaches uses wavelet transform alongside thresholding, primarily for ocular artifact removal [66,68,122] and instrumental artifacts [61], applied in both single-channel [61,66] and all-channel configurations [122]. In particular, for ocular artifacts, wavelets enable transient localization in the time–frequency domain by decomposing signals into frequency bands, allowing thresholding to be applied directly on the wavelet coefficients of low-frequency components for detection (for instance, Bahadur et al. (2024) [122] and Peng et al. (2013) [66]). Furthermore, Kaongoen et al. (2023) [61] employ stationary wavelet transform combined with ASR to decompose single-channel EEG signals into components of equal sample length, attempting to address challenges associated with the limited number of channels in low-density systems. The combination of ICA with thresholding is widely applied for ocular artifact removal [82] as well, and often also for muscular artifacts and PLN [75], cardiac [78], or movement artifacts [84], in single-channel [78], multi-channel [75], and all-channel configurations [82,84]. ICA separates statistically independent components, while thresholding identifies and removes those containing artifacts. Studies differ in the features used for thresholding and in the ICA variants adopted to enhance component separation, beyond channel specificity and targeted artifacts. For instance, Val Calvo et al. (2019) [82] employ wICA and Enhanced Automatic wICA (EAWICA), analyzing kurtosis and multi-scale sample entropy in the first case, and kurtosis together with Renyi entropy in the second one. Cheng et al. (2019) [78] applies the classic ICA approach, evaluating the autocorrelation of the extracted sources. In contrast, some workflows apply ICA without thresholding, as seen in Sweeney et al. (2012) [64] and Islam et al. (2020) [90], both addressing cable movement artifact detection, with the latter also targeting body and limb movements. Sweeney et al. (2012) [64] employ EEMD-ICA, adaptive filter and Kalman filter, according to the artifact, using manual detection based on signal shape, frequency, and amplitude. Islam et al. (2020) [90] use Infomax ICA, assessing independent components through topographic maps, spectral analysis, and autocorrelation in an all-channel framework. The FFT combined with thresholding is used in pipelines targeting muscular artifacts alongside ocular and movement artifacts [59] and also PLN [58]. In this context, FFT computes the spectral power distribution, while thresholding is applied to parameters such as average power [59] or high-frequency energy for calculating indices like kurtosis and Median Absolute Deviation (MAD) [58], with implementations in single-channel [58] and multi-channel [59] settings. In another case, Matiko et al. (2013) [65] apply Short-Time Fourier Transform (STFT). Five studies implement ASR-based pipelines for the ocular artifact [85], in combination with muscular artifacts [107], movement [94,123], or also with cardiac and instrumental artifacts [106]. In these methods, the decision rule typically involves thresholding based on the mean and standard deviation of the calibration signal, with differences arising from the features used. Kumaravel et al. (2021) [94] and Xiao et al. (2022) [106] apply traditional ASR, using PCA on sliding windows to compare component energy against calibration thresholds. Arpaia et al. (2022) [107] and Blum et al. (2019) [85] additionally test the Riemannian ASR variant, replacing PCA with PGA, and projecting data onto geodesic sub-manifolds. Finally, Arpaia et al. (2024) [123] propose MEMD as a preliminary step, allowing ASR to operate on the extracted IMFs. A unique case is presented in the study of Rosanne et al. (2019) [84], characterized by multiple algorithms for ocular and movement artifact removal using thresholding in an all-channel configuration, each paired with specific feature extraction methods. The evaluated pipelines include (i) ASR with short-window variance derived through PCA; (ii) Automatic Artifact Removal (AAR) based on Second Order Blind Identification (SOBI), assessing cross-correlation matrices and fractal dimensions to identify ocular components; (iii) SOBI-based wICA; (iv) wICA preceded by a wavelet decomposition step for IC extraction; and (v) Infomax ICA, using kurtosis, spatial average, variance difference, spatial eye difference, and discontinuity analysis. Studies using EMD and variants show different approaches for the removal of EEG artifacts. Occhipinti et al. (2022) [101] apply Noise-Assisted Multivariate EMD (NA-MEMD) to decompose multi-channel signals into IMFs synchronized across channels. The signals are considered free of muscular and movement artifacts when their power spectral density remains below a defined threshold. Chen et al. (2022) [100] integrate EMD with wavelet analysis through Empirical Wavelet Transform (EWT), subsequently applying DWT to the IMFs and comparing the pipeline with Maximal Overlap Discrete Wavelet Transform (MODWT)-Multiresolution Analysis (MRA) and CCA. In this case, six features are computed on each component and used within a one-class SVM to detect ocular, muscular, movement, and EMI artifacts. Finally, Narmada et al. (2023) [111] propose an EMD-DWT combination optimized using the Opposition Searched–Elephant Herding Optimization (OS-EHO) algorithm in order to enhance the efficiency of ocular, cardiac, and muscular artifact removal while preserving physiological EEG components. Thresholding is also applied in CCA-based algorithms for muscular artifact removal, as presented by Liu et al. (2019) [88] and Liu et al. (2021) [93]. In Liu et al. (2019) [88], CCA is combined with Fast Multivariate EMD (FMEMD) to extract IMFs and compute signal autocorrelation. A feature selection phase is then performed, randomly selecting between three and eight channels from the original trace for subsequent artifact removal stages. Liu et al. (2021) [93], instead, test three different variants: (i) CCA with autocorrelation coefficients evaluated across all channels; (ii) EEMD-CCA applied to IMFs in a single-channel setup; and (iii) MEMD-CCA for analyzing multivariate IMFs in a multi-channel configuration. Additionally, an adaptive RLS filter is tested on single-channel data with EMG reference, allowing evaluation of coefficients associated with EMG components by minimizing a weighted least squares cost function. Singular cases include Noorbasha et al. (2020) [91] and Jiang et al. (2023) [113], both addressing ocular artifact detection. Noorbasha et al. (2020) [91] apply Singular Value Decomposition (SVD) with MDL-based subspace recognition to identify and extract ocular components from single-channel EEG. Jiang (2023) employ VME to isolate mode functions, subsequently evaluated through thresholding for artifact detection. Additionally, some workflows integrate feature extraction methods as wavelet, FFT, and ICA, with supervised classifiers as SVM, k-Nearest Neighbors (kNN), and Random Forest for detecting movement artifacts [70], ocular and muscular artifacts [74], eye blinks [77], and, in some cases, also instrumental artifacts [83]. Studies vary in classifier selection, chosen according to dataset characteristics, and in the potential inclusion of a feature selection step before classification. For instance, Ingolfsson et al. (2022) [99] employ TPOT to select energy features derived from DWT and FFT, subsequently applying binary, multi-class, and multi-class multi-output classification. In a later study, Ingolfsson et al. (2024) [124] use TPOT during training for feature selection of similar energy features, combined with Minimal Cost-Complexity Pruning (MCCP) for embedded model construction. Moreover, Dey et al. (2020) [92] apply correlation analysis and a *t*-test for feature selection on time series extracted after windowing, followed by MLP-based classification of the analyzed windows. Finally, deep learning-based models commonly combine convolutional layers with fully connected layers using softmax or sigmoid activation functions for classification. This approach represents about 15% of the analyzed cases, targeting ocular and muscular artifacts [102,118,120,126], movement [119,121,125,132], or power line interferences [105]. Feature extraction relies on convolutional feature maps automatically generated by the convolutional layers, encoding complex patterns associated with both artifacts and physiological EEG activity, and enabling it to distinguish artifactual components automatically. Classification typically involves fully connected layers with softmax or sigmoid activations, without explicit thresholding using the argmax or the intrinsic sigmoid threshold for decision-making. Recent approaches employing autoencoder and UNet architectures, as proposed by Mahmud et al. and Saleh et al., implement clean signal reconstruction without a separate classification phase, integrating detection and removal within the reconstruction process. The majority of methods are applied in single-channel configurations, with Nair et al. (2025) [126] representing an exception by extending the algorithm to two channels simultaneously.

The following paragraph focuses on the hyperparameters associated with each of the methods under investigation. In wavelet-based methods, the mother wavelet type is most frequently Daubechies (approximately 60% of the wavelet studies), followed by Haar (about 30%), with decomposition levels most often between 5 and 7 (around 70% of the studies). ICA-based pipelines differ in the number of extracted components: in about 55% of cases, the number of ICs was set equal to the number of channels. ASR approaches reported different best cutoff values, most commonly in the range 4–9 (70% of ASR-based studies), but occasionally up to 25 (20%), with window lengths of 0.3–0.5 s in approximately half of the cases. For FFT and STFT methods, window functions such as Hanning in 60% of the cases, with FFT lengths most often set to 1024 points in the reported implementations, are specified. For CCA and EMD variants, the number of channels used ranges from 3 to 8 in about 40% of the cases, while the remaining studies employed all available channels; noise channels and the number of directions in E(M)EMD were reported in about 50% of these studies. In CNN models, kernel size is typically mono dimensional and small (≤3 in over 65% of cases), input segment length is most frequently 0.5–2 s (60%), with Adam or AdamW optimizers applied in over 80% of cases.

#### 3.4.1. ASR and Its Relevance for Wearable EEG Artifact Management

ASR is an algorithm used for removing high-amplitude artifacts, such as eye blinks, muscle activity, and movement-related artifacts, from EEG data [146,147]. Therefore, ASR appears promising for application with EEG wearable devices, allowing recordings to be performed during motor tasks. In fact, wearable EEG devices experience more frequent artifacts, largely because they operate in dynamic, everyday conditions such as locomotion [94,97]. In contrast to common considerations, the essence of ASR is not only based on the application of PCA itself but rather encompasses subspace reconstruction accounting for statistical deviations from a clean signal model. In this regard, Kim et al. (2025) [146] expect promising results from replacing PCA with ICA or other methods, although they have not tested them yet. In the first step, ASR identifies clean reference segments. The signal is divided into epochs and, for each epoch, a wise-channel root mean square (RMS) is computed. A z-score-based criterion is then applied across the RMS values of all channels, and an epoch is rejected only if its z-score falls outside the acceptance range in up to 7.5% of the channels. From the remaining clean epochs, covariance matrices are computed, and their median is used to define the reference matrix. The eigenvalue decomposition of the reference matrix provides the basis of the principal components for subsequent analysis. The data are then projected into the component space. Within the principal component space, ASR estimates rejection thresholds for each component. In particular, the RMS of each principal component is computed, and an acceptance interval, depending on its mean and variance scaled by a sensitivity parameter “k”, is defined. When applied to raw EEG data, each new epoch is projected into the same principal component space. For each epoch, only those principal components whose RMS falls within the predefined acceptance interval are retained. The inverse transformation of the retained components reconstructs the artifact-cleaned signal.

Modified implementations of the ASR algorithm have been adopted in several studies. Blum et al. (2019) [85] and Arpaia et al. (2022) [107] employ the Riemannian Artifact Subspace Reconstruction (rASR) approach. rASR uses Riemannian geometry to process covariance matrices, which are symmetric positive-definite (SPD) and reside in a curved manifold. rASR computes the Karcher mean (Riemannian equivalent of the arithmetic mean) to average covariance matrices. It also replaces PCA with Principal Geodesic Analysis (PGA) to generalize dimensionality reduction to the curved manifold of SPD matrices. Another variant of ASR, proposed by Xiao et al. (2022), applies a Fast Fourier Transform (FFT) and uses signal energy within specific frequency bands for calibration instead of RMS. This ensures that the reference data are relevant to the subsequent analysis by focusing on frequency bands of interest. Conversely, Kumaravel et al. (2023) [115] develop a method leveraging accelerometer data from Inertial Measurement Units (IMUs) to replace the conventional calibration step of the ASR algorithm. This approach is based on the principle that the magnitude of accelerometer data directly correlates with motion artifacts in the EEG signal. By identifying outlier segments in the accelerometer data, the method can effectively detect contaminated EEG data, providing a practical solution for real-time analysis in mobile EEG systems. In addition, several studies have proposed combined approaches integrating ASR with other methods, aiming to enhance its performance beyond the standard implementation. Rosanne et al. (2019) [84] explored two such strategies: (i) ASR combined with Automatic EEG artifact detector based on the joint use of spatial and temporal features (ADJUST), where the components are automatically identified as artifactual and subsequently removed after applying ICA, and (ii) ASR combined with Wavelet-ICA, which applies wavelet-based thresholding to ICA components to effectively suppress high-amplitude artifacts such as eye blinks. Kaongoen et al. (2023) [61] further introduced other method combinations. By first decomposing single-channel EEG signals using singular spectrum analysis (SSA), ensemble empirical mode decomposition (EEMD), or wavelet transform (WT), they generated multiple subcomponents that were then processed with ASR. This design adapted ASR to a single-channel context, highlighting how signal decomposition can expand ASR applicability to scenarios with limited spatial information. Finally, Arpaia et al. (2024) [123] proposed multivariate empirical mode decomposition (MEMD)-ASR, where MEMD provides a channel-consistent decomposition into scale-aligned intrinsic mode functions (IMFs) before ASR is applied. Unlike approaches that decompose channels individually, MEMD preserves inter-channel relationships and avoids the loss of information introduced by transformations, thus supplying ASR with input that is both higher in dimensional integrity and better aligned across channels.

Focusing on the stand-alone use of ASR, its effectiveness is highly sensitive to the values of user-defined parameters. Two main parameters are crucial for ASR [146]: the window length and the cutoff threshold *k*. A typical window length is 0.5 s; however, optimal performance is typically found with *k* values between 10 and 30. An excessively long window may fail to capture transient artifacts when using RMS, whereas an overly low *k* value can lead to the removal of valid neural signals, and an overly high *k* value may result in insufficient artifact suppression. The parameter and configurations used in the collected studies are *k* = 7 and window length = 0.5 s (Rosanne et al. (2019) [84]); *k* = 1 and window length = 0.3 s (Blum et al. (2019) [85]); k∈[1,100] (Kumaravel et al. (2021) [115]); *k* = 15, 25 for ASR, and *k* = 2, 5 for rASR (Arpaia et al. (2022) [107]); k∈[1,30] and window length = 0.5 s (Kaongoen et al. (2023) [61]); *k* = 7–12 and window length = 0.5 s (Arpaia et al. (2024) [123]). In particular, Rosanne et al.’s (2019) [84] results indicate that ASR-ADJUST was the most effective under medium-movement conditions, while ASR-wICA performed best during high movement. Blum et al. (2019) [85] demonstrate ASR produces overcorrection with *k* = 5), which required less aggressive settings. The alternative rASR, using a lower *k*, preserved Evoked Potential morphology while being computationally more efficient (82% reduction in runtime compared to ASR), highlighting that lower *k* values can be advantageous in low-amplitude, visually evoked paradigms. Kumaravel et al. (2023) [115] explore *k* across conditions of ocular and movement contamination in Steady-State Visual Evoked Potentials (SSVEP) paradigms, performing ASR by (i) removing artifacted segments (Removal mode) and (ii) reconstructing the signal (Correction mode). Optimal *k* values were frequency- and mode-dependent: *k* = 10–20 for 2–4 Hz and *k* = 15–40 for 8 Hz. Moreover, the Removal mode of the ASR required slightly higher *k* (≤8) to avoid excessive rejection of data segments, while Correction mode allowed lower thresholds. Importantly, IMU-ASR achieved comparable performance to standard ASR while reducing computation time by ∼94%. In low-channel configurations, Arpaia et al. (2022) [107] compare ASR (with *k* = 15 and 25) and rASR (with *k* = 2 and 5) against ICA and PCA. Results show that ASR preserved baseline EEG activity more effectively than ICA or PCA, which either distorted clean signals or failed in few-channel settings. While rASR was computationally efficient, its low *k* values tended to excessively modify clean EEG. In the study by Kaongoen et al. (2023) [61], performance peaks at very low cutoff thresholds (*k* = 5 for a movement dataset; *k* = 4 for an ocular dataset) and deteriorates with higher values. Finally, Arpaia et al. (2024) [123] report optimal performance at *k* = 9 with 4 channels. Compared to ASR alone, MEMD-ASR avoids overcorrection.

### 3.5. Artifact Detection vs. Artifact Category Identification Strategies

**Figure 5:**
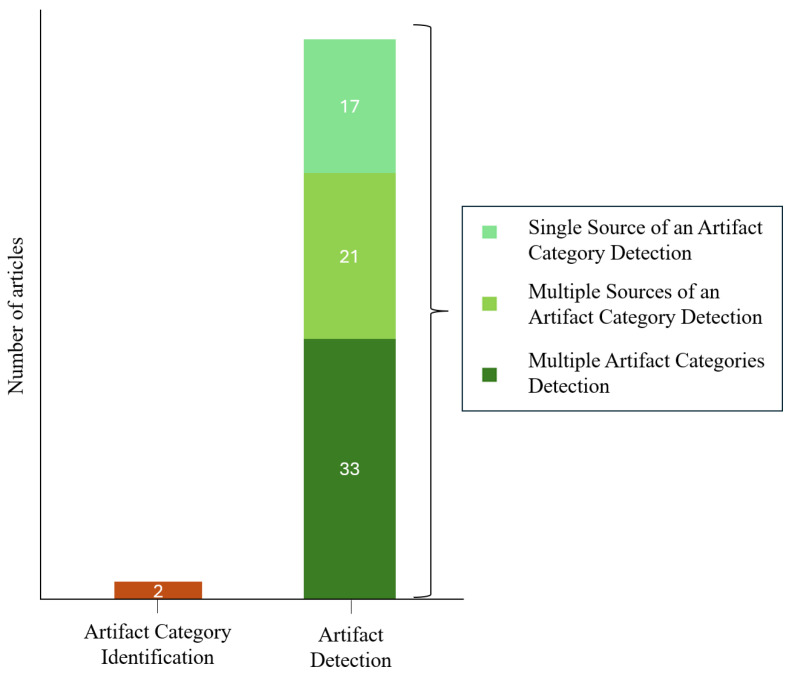
Number of articles focusing on artifact detection strategies (green) and artifact category identification strategies (orange) across the reviewed studies. Detection strategies are further classified based on their robustness, defined as the extent of artifact sources used for validation. Only two studies attempt to identify the specific category or source of artifacts.

The artifact category discrimination capability of the reviewed methods was assessed according to the level of detail in artifact source differentiation. As illustrated in Figure 5, the subsequent strategies were identified: (i) approaches addressing multiple artifact categories in a source-unspecific manner (33 studies); (ii) methods detecting multiple sources within the same artifact category (21 studies); (iii) methods targeting a single artifact source (17 studies); (iv) methods attempting to identify distinct artifact sources (2 studies). Most studies in the first category adopt a binary approach, distinguishing clean and contaminated signals without specifying the artifact category present in the affected epochs. For instance, Ingolfsson et al. (2022) [99] propose a Multi-class Multi-output Classification (MMC) system capable of detecting twelve artifact sources, including ocular, instrumental, muscular, and movement-related artifacts. The system labels each time window and channel as either clean or contaminated, without differentiating between artifact categories. Moreover, Li et al. (2023) [116] demonstrate the generalizability of their detection method across ocular, muscular, and movement artifacts and show that segmenting the signal into clean and contaminated portions enhances artifact removal performance in terms of accuracy and selectivity. Grosselin et al. (2019) [83] propose an algorithm representing an early step toward artifact category identification. Specifically, the study evaluates EEG signal quality from single-channel wearable devices by distinguishing among clean signals, broadly contaminated signals (ocular and movement-related), and muscular artifacts (masseter and temporalis), thereby enabling the identification of specific muscular artifact sources. Zhang et al. (2021) [58] and Inoue et al. (2019) [59] stand out as the only studies fully meeting the criteria of the fourth category. Inoue et al. (2019) [59] develop an approach relying on amplitude thresholds and spatio-temporal symmetry to identify blinks, eye movements, and muscular artifacts. Zhang et al. (2021) [58] propose a two-stage pipeline for single-channel EEG, separately identifying and removing ocular, muscular artifacts, and PLN. In particular, the first stage detects the artifact category, and the second applies targeted removal, preserving clean signal segments.

## 4. Discussion

This review investigated four main aspects in the literature: the specific challenges of artifact management in wearable EEG systems (RQ-I), the algorithms used for artifact detection and classification (RQ-II), the parameters (RQ-III), and the metrics and reference signals (RQ-IV) used to assess algorithm performance. In the following sections, the discussion is organized into three paragraphs for RQ-I, RQ-II, and RQ-III, together with RQ-IV. Finally, in Section 4.4, emerging perspectives and limitations of the present review are reported.

### 4.1. Specific Challenges of Artifact Management in Wearable EEG Systems

The literature shows a notable delay in addressing the unique challenges introduced by wearable EEG technologies. Only a limited number of studies explicitly consider the heightened complexity posed by these systems, particularly in managing movement-related artifacts. Such artifacts are more frequent and severe in wearable EEG due to their operation in dynamic, real-life settings (e.g., walking, running) [94,97]. They are characterized by high amplitude, non-stationarity, and broad spectral content that overlaps with neural signals, severely limiting the efficacy of traditional filtering techniques. Artifacts from cable motion are especially problematic due to their transient, irregular patterns and lack of time-locking with physical movements [148]. Additionally, the literature underrepresents the impact of electrode type. Dry electrode systems, increasingly used in wearable EEG, are more prone to artifacts due to the absence of conductive gel, which in wet systems helps stabilize the electrode–skin interface and maintain low impedance [56,67]. A further gap concerns the limited use of auxiliary signals for artifact detection and removal in wearable EEG. In traditional EEG systems, additional channels such as EMG and EOG are commonly employed to monitor and subtract specific sources of contamination. However, these solutions are less compatible with wearable applications. In the context of wearable EEG, more suitable alternatives include sensors such as accelerometers, microphones, and other miniaturized devices. Although some studies have begun to integrate these wearable-compatible sensors (e.g., IMUs for motion detection [115], ETI for impedance monitoring [101], microphones for vibration localization [101]), their adoption remains limited. A notable exception within the broader scientific consensus is the work of Sweeney et al. (2012) [64], who employed gold-standard, non-wearable equipment to preserve a physiological reference signal recorded near an artifact-contaminated site. The artifact was induced through deliberate cable movement. His study stands out for its explicit recognition of the unique challenges posed by wearable EEG systems, particularly the difficulty in distinguishing signal from artifact under the novel, low-SNR conditions that characterize real-world, mobile recordings.

In addition, several artifact sources specific to wearable EEG remain largely unaddressed in the reviewed literature. For example, fluctuations in the reference channel-to-ground potential [149], such as those caused by foot movement, can introduce noise at the electrode level. While this issue is typically minimized in wired systems sharing a common ground with the ADC, it may be exacerbated in wireless configurations. Similarly, although wireless systems offer increased immunity to conducted electrical noise, they remain vulnerable to radiated interference, including electric arcs or transient surges from nearby electronic devices. Exposure to common-mode noise, such as environmental EMI, is also more critical in wearable contexts [150,151]. While powerline noise is occasionally considered, other radiated or device-induced interferences are rarely explored. This is particularly problematic given the uneven distribution of noise across electrodes, especially with dry sensors, which undermines the effectiveness of conventional solutions like the Driven Right Leg (DRL) circuit [152]. In such conditions, generating a stable counter-phase signal becomes unreliable and may even amplify noise. Another neglected issue concerns impedance fluctuations due to sweating, especially in dry electrode systems [153]. These variations can cause slow signal drifts that standard preprocessing pipelines struggle to correct. Finally, the application of conventional artifact removal strategies for low-density and single-channel configurations is not easily applicable also due to the reduced scalp coverage [60,61,64,76,91,109].

### 4.2. Algorithms for Artifact Detection and Classification

In general, the algorithmic pipelines described in the literature include two main stages: artifact detection/identification and artifact removal. Most detection modules perform a binary identification of artifact presence, without distinguishing between different categories or sources. Some algorithms are validated on multiple artifact types, which shows a certain level of generalization capability. However, this generalization applies only to the detection phase and does not extend to the identification of artifact types. For this reason, their usefulness remains limited when more detailed information is needed for further processing. Only two studies attempt to implement a classification of artifact category or source [58,59]. In the removal phase, several algorithms are designed and tested assuming that the artifact type is already known. This allows for good performance in controlled scenarios. However, it limits their applicability in real-world conditions, where artifacts are often mixed and not labeled in advance. These algorithms are rarely tested on signals that reflect the variability of real physiological recordings, which reduces their practical value. At the same time, the high specificity of these algorithms in removing well-defined artifact types makes them promising for modular pipelines. A two-step architecture, with (i) source identification followed by (ii) targeted artifact removal, could be an effective approach for wearable EEG applications [54,55,57]. Among the most robust approaches for binary artifact detection, Ingolfsson et al. (2022) [99] propose an MMC system capable of detecting twelve artifact sources, including ocular, instrumental, muscular, and movement-related artifacts. The system labels each time window and channel as either clean or contaminated, without differentiating between artifact categories. Furthermore, Li et al. (2023) [116] not only demonstrate the generalizability of their detection method across EOG, EMG, and movement artifacts but also show that segmenting the signal into clean and contaminated portions improved artifact removal performance in terms of both accuracy and selectivity. Some studies represent early steps toward effective artifact category identification. For example, Grosselin et al. (2019) [83] assessed EEG quality from single-channel wearable devices, distinguishing clean, generically contaminated (ocular and movement), and muscle-contaminated signals (masseter and temporalis), thus providing the basis for specific muscular artifact sources identification.

Zhang et al. (2021) [58] and Inoue et al. (2019) [59] increase the number of artifact categories targeted for identification. In particular, Inoue et al. (2019) [59] introduce a method for distinguishing blinks, eye movements, and muscular artifacts using amplitude thresholds and spatio-temporal symmetry criteria. However, the procedure is not clearly described, and the algorithm has been tested on only one subject, limiting the assessment of the method’s actual discrimination capability. On the other hand, Zhang et al. (2021) [58] propose a two-stage pipeline for the detection and removal of ocular, muscular artifacts, and powerline noise in a single-channel EEG system. The identification stage relies on a cascade of three dedicated detection blocks, each targeting specific artifact categories and sources. When a block successfully detects artifacts within its scope, the identification process terminates; otherwise, the signal is forwarded to the next block. This work provides the most promising results for artifact category identification, although it does not perform source-level analysis for ocular and muscular artifacts. Many proposed detection methods are developed and validated on signals containing specific artifact categories or sources. Although these approaches may not generalize well when processing real-world signals, they provide a valuable foundation. After assessing their behavior against artifact categories beyond those used for validation, such methods could serve as building blocks for designing cascade-based identification systems composed of specialized detection modules. Wavelet transforms and ICA, both using thresholding as the artifact decision rule, are commonly employed to manage ocular and muscular artifacts. ASR-based pipelines, often integrating PCA and thresholding, are widely applied to address ocular, movement, and instrumental artifacts. Supervised classification approaches (e.g., SVM, kNN, Random Forest) are typically coupled with feature extraction to detect muscle and motion artifacts.

The gap in artifact categorization within the selected studies likely stems from the cultural background of the authors of the papers. Predominantly associated with the disciplinary fields of computer science and engineering, they tend to maintain an approach focused on implementing automated removal solutions, favoring a black-box approach that prioritizes algorithmic efficacy over an in-depth understanding of the underlying neurophysiologic phenomena. This is evidenced by the scarcity of comprehensive databases mapping the full spectrum of artifact types, contrasting with existing datasets that are typically limited to single artifact classes (see Table 2).

Regarding the epoching for the algorithm application, most studies do not provide a clear rationale for the choice of a specific length. However, Jayas et al. (2023) [110], using machine learning techniques, explore the optimal segment length by testing durations of 1 s, 2 s, and 5 s, with 5 s epoch yielding the best performance.

In addition, several studies involve adapting techniques originally developed for high-density EEG systems to low-channel configurations [60,61,85,104,107,109,115,116]. However, authors do not always offer a critical consideration of using these algorithms in low-channel settings. For instance, ICA is often applied in low-density configurations without discussing the limitations introduced by reduced spatial coverage [82,87]. Indeed, source separation techniques, as ICA, are particularly effective when applied with 32 or more electrodes [48]. Nonetheless, Arpaia et al. (2022) [107] experimentally demonstrated the decline in performance of ICA using fewer than eight channels, suggesting that methods like ASR may be better suited for low-cost and wearable EEG systems. Instead, Cheng et al. (2019) [78] proposed a strategy to overcome the low-channel constraint by using SSA to create artificial multi-channel datasets from single-channel signals, enabling ICA application even in the absence of real multi-channel data.

### 4.3. Performance Assessment Parameters, Metrics, and Reference Signals

The results regarding the performance assessment of the algorithms are not exclusively associated with either the detection or the removal phase but rather refer to the combined sequence of both phases. Three main categories of reference signals are used: (i) clean signals, (ii) artifact signals, and (iii) physiological signals. Clean signals can be simulated or real. In the case of real clean signals, these are typically obtained through two approaches. The first is an ex ante strategy, where the subject is instructed to remain still or relaxed during data acquisition in order to minimize artifact generation. However, this does not ensure the complete absence of contamination. The second is an ex post strategy, where clean segments are selected after acquisition. This can be performed using traditional artifact removal algorithms or through visual inspection, which introduces subjectivity and depends on the user’s level of expertise. In the case of simulated clean signals, most approaches rely on the random generation of signals with partial attempts to model neurophysiological patterns, although they only partially capture the dynamic and nonlinear complexity of typical physiological EEG activity [66,68,71]. Clean signals are mainly used to assess the accuracy. In this review, accuracy is defined according to the International Vocabulary of Metrology (VIM3) [154] as the closeness of agreement between a measured value and a true value of a measurand. As a result, in this context, the reference signal cannot be considered a true value in a narrow metrological sense.

Some studies adopt artifact signals as a reference for detection performance assessment. Typically real and not simulated, reference artifact signals allow for the computation of standard classification metrics such as classification accuracy (the percentage of correct predictions out of the total predictions). Physiological signals, representing partially contaminated real recordings, are also used as references. These signals are particularly useful for evaluating selectivity, i.e., the algorithm’s ability to preserve brain activity while removing artifacts [65,66].

Further parameters are assessed independently of the EEG signal. Hardware efficiency is rarely evaluated, despite its relevance for real-time and embedded implementations of the algorithms in ecological conditions. For instance, the related metric power consumption occurs in only three studies [92,106,132]. Remarkably, the metrics computational burden [132] and silicon area [122] are each considered in only one study.

An analysis providing the main findings, with a focus on robustness, metrics, and hyperparameter sensitivity of the proposed methods is reported below in order to deeply assess the challenges of dry electrodes or movement-related artifacts in wearable EEG scenarios. The analysis groups the studies according to the main artifact category focused on, namely ocular, muscular, and movement artifacts.

With respect to ocular artifacts recorded with dry electrodes, most studies rely on wavelet-based or multicomponent decomposition methods. Early approaches (Matiko et al. (2013) [65], Peng et al. (2013) [66], Zhao et al. (2014) [68]) demonstrate moderate success but are highly sensitive to the choice of mother wavelet, number of decomposition levels, and windowing parameters, limiting reproducibility across datasets. More recent work, such as the MEMD-ASR approach (Arpaia et al. (2024) [123]), tests different cutoff thresholds (k between 7 and 12; k = 9 resulted as the best value) and multivariate empirical mode decomposition, showing improved robustness, especially with few-channel dry systems. Despite these advances, the lack of standardized performance metrics (e.g., SNR vs. CC vs. RRMSE) prevents direct numerical comparisons, though qualitative evidence suggests that ASR-based pipelines offer superior preservation of neural signal integrity.

For muscular artifacts recorded using dry electrodes, Grosselin et al. (2019) [83] and Arpaia et al. (2024) [123] adopt different approaches, though the two studies remain only partially comparable due to methodological and metric differences. Grosselin et al. (2019) employ a classification-based strategy, reporting high accuracy (92.2 ± 2.2%) and SNR-dependent performance (e.g., 99.8% accuracy at <0 dB, dropping to 43.1% at 10 dB). This highlights the potential of supervised learning but also its dependence on SNR levels and training data quality. By contrast, Arpaia et al. (2024) introduce the MEMD-ASR pipeline, tailored for non-stationary artifacts and robust even with very few channels.

Regarding movement-related artifacts using dry electrodes, classical adaptive filtering approaches (Mihajlović et al. (2014) [67]) or ICA-based pipelines (Islam et al. (2020) [90]) show improvements in SNR and coherence but require either additional reference channels or careful manual selection of independent components to avoid removing neural information. Sweeney et al. (2012) [64] demonstrate that integrating accelerometer references into Kalman or EEMD-ICA pipelines yields substantial SNR gains, though at the cost of complexity and processing time. Mihajlović et al. (2014) [67] introduce impedance-based filtering (ETI-MCAF), efficient in real time but less reliable at low frequencies (<2 Hz). Also, M-mDistEn (Aung et al. (2021) [97]) shows high performance but requires parameter tuning. More recently, IMU-ASR (Kumaravel et al. (2023) [115]) and MEMD-ASR (Arpaia et al. (2024) [123]) achieved robust results with simpler configurations and improved computational efficiency. The IMU-ASR framework (Kumaravel et al. (2023) [115]) represents a significant step forward, replacing computationally expensive calibration with inertial measurements, thus reducing processing time (∼94%) while maintaining performance. Optimal k thresholds (10–40, depending on frequency and removal vs. correction mode) allow fine-tuning of artifact suppression without excessive signal loss, making this method particularly suitable for real-time or wearable applications. In Rosanne et al. (2019) [84], combinations of ASR and other methods achieved better results than ADJUST, Wavelet-ICA, Harvard automated processing pipeline for electroencephalography (HAPPE), AAR, or AAR+wICA: ASR-ADJUST performed best under medium movement and ASR-wICA under high movement during mental workload conditions. Similarly, MEMD-ASR pipelines (Arpaia et al. (2024) [123]) demonstrated resilience even in minimal-channel settings (2–4 electrodes).

Instead, several studies evaluate artifact suppression in the context of movement artifacts and cable-induced instrumental artifacts across wet and dry electrode setups. Methods such as Kalman filtering or EEMD-ICA (Sweeney et al. (2012) [64]) deliver strong SNR improvements (∼9–10 dB), albeit with high computational cost and dependency on reference accelerometers. ASR-based hybrid pipelines (SSA-ASR and WT-ASR) (Kaongoen et al. (2023) [61]) reach the highest Δ SNR (∼15 dB) against ICA, SSA, WT, EEMD, with low k thresholds (4–5), underscoring the sensitivity of ASR tuning. Similarly, MEMD-ASR (Arpaia et al. (2024) [123]) and rASR (Blum et al. (2019) [85]), against traditional ASR, prove highly effective, particularly in preserving morphological features and operating efficiently in low-channel systems. These findings indicate that ASR, especially when integrated with complementary decomposition methods (such as SSA, WT, MEMD), offers a flexible and computationally efficient solution for artifact suppression.

Across all scenarios, ASR consistently outperforms or matches alternative methods (ICA, PCA, HAPPE), particularly in conditions with movement artifacts, limited channels, or real-time requirements. Parameter tuning, especially the cutoff threshold k, is critical for balancing artifact suppression and signal preservation. Empirically, moderate k values (4–9) perform best for ocular or moderate movement artifacts, while higher thresholds (∼10–25 or up to 40 for high-frequency SSVEP tasks) are preferable in removal modes or high-noise conditions. In fact, the selection of ASR parameters could depend on the specific task being performed. For instance, in [155], it is highlighted how the choice of ASR cut-off parameter should be adapted to the level of movement-related artifacts in the EEG data. Specifically, tasks that induce greater movement contamination tend to require lower cut-off values to achieve effective reconstruction, comparable to the amount of data typically removed during manual cleaning. For example, in tasks such as the single-leg stance and n-back, the optimal cut-off ranges align with values previously reported in the literature, such as the 5–7 range suggested by Mullen et al. (2015) [156] for BCI applications with dry electrodes and the 20–30 range adjusted by Chang et al. (2019) [157] for EEG data recorded during simulated driving. However, for the walking task, lower cut-off parameters are necessary, likely due to the higher level of movement artifacts compared to less dynamic conditions such as simulated driving. This evidence underscores the importance of task-specific calibration of ASR parameters to ensure optimal artifact removal while preserving neural signal quality. Hybrid approaches, such as MEMD-ASR or IMU-ASR, enhance robustness and reduce computational load, making ASR-based pipelines suitable for wearable and mobile EEG applications. Moreover, ASR maintains stability in minimal-channel dry systems, where ICA often distorts clean signals. The main limitation across the literature is the lack of standardized metrics, which hinders quantitative comparisons across methods and datasets. Moreover, many algorithms exhibit significant hyperparameter sensitivity, making their performance highly dependent on dataset characteristics and requiring careful tuning.

### 4.4. Emerging Directions and Limitations

In relation to the management of movement artifacts, a study published after the closing date of the present literature search [146] proposed two new approaches based on ASR: (i) ASR-Density-Based Spatial Clustering of Applications with Noise (DBSCAN) and (ii) ASR-Generalized Extreme Value (GEV). These new approaches prevent short, burst-like movement artifacts (typical of intense real-world motor tasks) from being included in the reference data. They are based on redefining the calibration phase by sampling each individual data point instead of the RMS value over a window, resulting in less skewed calibration distributions with smaller related threshold values (k values between 3 and 5). Although these approaches have not been tested on wearable EEG, they could represent an alternative approach, for example, in cases where IMU is not available.

Moreover, recent deep learning–based approaches for EEG artifact handling reveal promising advances. Generative adversarial networks with recurrent generators and convolutional discriminators [105], as well as transformer-based models, have been applied to both multi-channel [104] and single-channel [118,120] configurations, demonstrating effective suppression of ocular and muscular artifacts and achieving satisfactory performance even when the number of available channels is reduced. For multiple artifact types, different strategies have emerged, encompassing sequential autoencoders [109], dual-branch fusion networks [60], and segmentation–denoising frameworks [116]. Wavelet-enhanced generative adversarial networks have further improved adaptability, though with higher computational cost [111]. In wearable contexts, compact autoencoders have enabled real-time denoising on embedded hardware [125], while hybrid systems combining convolutional networks with least-mean-square filtering have proven effective on two-channel EEG [126]. Clinical studies in neonatal EEG also confirm that semi-supervised convolutional networks can capture diverse contamination sources with reduced channels [119,121]. The gradual transition toward deep learning has yielded consistent performance gains, as highlighted in the comparative study by O’Sullivan et al. (2023) [119], where convolutional neural networks outperformed traditional thresholding and machine learning methods. In this work, the authors compared three strategies for artifact detection in neonatal EEG recorded with a nine-channel device: a threshold-based digital signal processing method combined with a compact neural network, a machine learning classifier based on random convolutional kernel transformations, and a fully convolutional deep learning architecture. When assessing all artifact types jointly, the convolutional neural network achieved higher results in typical classification performance metrics, such as Area Under the Curve (AUC) (+20%), Matthews Correlation Coefficient (MCC) (+36%), and sensitivity (+100%) compared to the threshold-based approach, and modest gains over the machine learning classifier (AUC +4%, MCC +6%, sensitivity +9%). For individual artifact categories, the machine learning and threshold-based methods occasionally outperformed the convolutional model, particularly for movement and muscular artifacts, where the machine learning classifier achieved up to 7% higher AUC and the threshold-based approach obtained more than 150% higher MCC. These results highlight that while complex deep networks generalize better across heterogeneous contaminations, simpler and more interpretable algorithms may be more effective for specific artifact categories that exhibit clear and easily detectable signatures. In general, deep learning approaches have achieved very high performance in controlled settings; however, they remain black-box systems, offering limited explainability and uncertain generalization to real-world conditions, especially when encountering artifact types not represented in the training data.

Some limitations of this review have to be acknowledged. Firstly, a quantitative meta-analysis was not possible due to the heterogeneity of methodologies, electrode configurations, experimental conditions, and outcome measures across the included studies. Moreover, the inclusion criteria considered only studies involving EEG devices with no more than 16 channels, dry or semi-wet electrodes, or hardware efficiency evaluation [47]. This may have excluded systems that do not fully align with these criteria, especially as far as the number of electrodes is concerned, but share key characteristics of portable EEG devices. Finally, the reported performance results were not evaluated in relation to the quality of the employed datasets. In particular, the use of simulated data in some studies and the limited sample sizes in others may limit the results’ generalizability.

## 5. Conclusions

This review systematically investigated methods for artifact detection and artifact category identification for EEG signals acquired using wearable devices, addressing artifact sources, artifact detection and removal algorithms, assessment parameters and metrics, and the corresponding reference signals. Artifacts in wearable EEG exhibit specific characteristics due to operational conditions such as the use of dry electrodes and the allowance of free movement. Additionally, their management poses unique challenges owing to the reduced scalp coverage. Although interest in wearable EEG has grown significantly in recent years, this trend has not been matched by a corresponding emphasis on artifact management in such setups. The majority of the papers propose a pipeline including both artifact detection and artifact removal phases. Only two studies address artifact category identification, possibly enabling the application of ad hoc strategies. Nonetheless, a trend of association between specific artifact and specific algorithms employed was observed, possibly useful to develop combinations of algorithms to perform identification. Wavelet Transforms and ICA, often using thresholding as a decision rule, are among the most frequently used techniques for managing ocular and muscular artifacts. ASR-based pipelines are widely applied for ocular, movement, and instrumental artifacts. Deep learning architectures are emerging as powerful alternatives, particularly for muscular and motion artifacts, and show promise for real-time implementation despite current limitations in online deployment. In addition, signals provided by auxiliary sensors (e.g., IMUs) remain underutilized, although they represent a key factor in improving artifact detection and removal in wearable EEG systems, particularly under ecological conditions. As far as the algorithm performance assessment is concerned, three main categories of reference signals are identified: (i) *clean*, (ii) *artifact*, and (iii) *physiological* (partially contaminated). Accuracy resulted as the most implemented assessment parameter, reflecting similarity to the *clean* reference. Instead, selectivity is assessed by adopting *physiological* signal as reference.

This review highlights that artifacts remain an overlooked topic in the wearable EEG literature, particularly regarding artifact category identification. This limitation affects artifact removal algorithms, which are developed and validated on specific artifact types with unknown performance on other artifacts or signals with heterogeneous artifact patterns. This review emphasizes existing contributions targeting artifact category identification and provides a mapping of removal algorithms validated on specific artifact types. A clear association emerges between artifact type and removal method, enabling the selection of appropriate techniques based on previously identified categories. Finally, a comprehensive survey of public datasets is also included to support standardization in artifact management for wearable EEG.

## Figures and Tables

**Table 1:** Acquisition setup and performance assessment methods of the algorithms proposed by the collected studies. The acquisition setup includes the experimental sample and task and the EEG channel setup. The signal processing methods refer to the algorithm employed, the specific artifact addressed, the reference signal, as well as the performance parameters and metrics with their corresponding results (reported in parentheses). When a study compared multiple algorithms, only the nomenclature and results of the best-performing one are reported. Regarding focused artifacts, a citation is provided only when the artifact is taken from a public dataset. The reported algorithms cover both the artifact detection and removal phases. S.N.S. = Source Not Specified, used when the artifact category is indicated without explicit information on its source. R = real recordings; S = simulated signals; SS = semi-simulated signals. “Not Applicable” (n.a.) indicates parameters not relevant in the context (e.g., acquisition setup for simulated data). “Not Reported” (n.r.) refers to parameters relevant but unspecified by the authors (e.g., participant sex). “Not Considered” (n.c.) refers to parameters relevant but not used in the study (e.g., absence of pre-processing). An explanatory table for other technical acronyms is recommended at the end of the document.

Article	Focused Artifact Category (Source)	Experimental Sample	Task Description	Channel Setup No. & Type (Location)	Reference Signal	Assessment Parameters	Assessment Metrics (Results)	Algorithm
Sweeney et al. (2012) [64]	Instrumental (Cable movements) [64]	**R**: 6 subjects 4 trials × 540 s	Resting-state	2 n.r. (Fpz, Fp1)	No contaminated channel	Accuracy	SNR (Δ SNR: (a) 5.1 dB; (b) 9.7 dB; (c) 8.9 dB), Correlation (improvement rate: (a) 37.66%; (b) 83.13%; (c) 76.5%)	(a) Adaptive Filter; (b) Kalman Filter; (c) EEMD-ICA
Matiko et al. (2013) [65]	Ocular (Eye blinks)	**R**: n.r. 60 trials × 1 s	n.r.	1 dry (Fp1)	Raw EEG	Selectivity	CC (improvement rate: 30.56%)	MCA based on STFT
						Operational speed	Latency (26.90 ms)	
Peng et al. (2013) [66]	Ocular (Eye movements, blinks)	**S**: 50 trials × 20 s	n.a.	1 n.a. (n.a.)	Initial EEG	Accuracy	MSE (0.00531), MAE (frequency: δ = 0.02233, θ = 0.01436, α = 0.00382, β = 0.00055; time: 0.00531)	DWT + ANC
		**R**: 25 subjects 1 trial × 120 s	Resting-state	3 dry (Fp1, Fp2, Fpz)	Raw EEG	Selectivity	Frequency domain correlation (numerical values n.r.)	
						Operational speed	Latency (numerical values n.r.)	
		**R**: 22 subjects 1 trial × 40 s	Resting-state	n.r.	Raw EEG	Selectivity	Frequency domain correlation (numerical values n.r.)	
Mihajlovic et al. (2014) [67]	Movement (Head)	**R**: 6 subjects 3 trials × 60 s	Motor tasks	4 dry (C3, C4, Cz and Pz)	EEG baseline	Accuracy	Spectral Score (reduction rate: ∼60–70%), Distribution Score (reduction rate: ∼70–80%)	BPF + leaky least-mean square MCAF
Zhao et al. (2014) [68]	Ocular (Eye blinks)	**S**: 50 trials × 30 s	n.a.	1 n.a. (n.a.)	Initial EEG	Accuracy	MSE (0.6443), MAE (δ: 0.2501; θ: 0.1545; α: 0.0975; β: 0.0174)	DWT + APF
		**R**: 20 subjects 1 trial × 120 s	Resting-state	3 dry (Fp1, Fp2, Fpz)	EEG baseline	Selectivity	Frequency domain correlation (numerical values n.r.)	
						Operational speed	Latency (5000 points, 1 s)	
Majmudar et al. (2015) [69]	Ocular (Eye blinks)	**R**: 3 subjects 1 trial × 45 s	Resting-state	2 wet (Fp1, Fp2)	Raw EEG	Selectivity	TFA (numerical values n.r.), MSC plot (f > 16 Hz: ∼1; f < 16 Hz: <1), CC (0.39 ± 0.25), MI (0.91 ± 0.12)	Algebraic approach + DWT
						Operational speed	Latency (improvement rate: ∼25%)	
Kim et al. (2015) [70]	Movement (Body, limb)	**R**: 5 subjects 1 trial × 300 s	Resting-state; dual-task	14 wet (AF3, F7, F3, FC5, T7, P7, O1, O2, P8, T8, FC6, F4, F8, AF4)	PSD at SSVEP and P300 frequency	Accuracy	SNR (Δ SNR: 0.26 ± 0.11 (SSVEP); 0.07 ± 0.10 (P300))	Fast ICA + Kalman filter + SVM
Rahman et al. (2015) [71]	Ocular (Eye blinks)	**S**: n.r.	n.a.	1 n.a. (n.a.)	Initial EEG	Accuracy	SNR (20.23 dB), MSE ($4.60\times 10^{-6}$)	SG filter + ANFIS
		**R**: n.r. 1 trial × 55 s	Resting-state	14 n.r. (only FP1 is reported)	EOG	Accuracy	SNR (16.98 dB), MSE ($3.39\times 10^{-5}$)	
						Selectivity	CC (measured Fp1/ estimated Fp1: 0.1478; measured EOG/ estimated eye blink: 0.9899)	
D’Rozario et al. (2015) [72]	Ocular (Eye movements, blinks); Muscular (S.N.S.); Movement (S.N.S.)	**R**: 24 subjects 2–4 trial × 28,800 s	Sleep	(a) 6 wet (C3, C4, Fz, Cz, Pz, and Oz), (b) 5 wet (C3, Fz, Cz, Pz and O2)	Artifacts identified by visual inspection	Classification Performance Metrics	Cohen’s kappa (0.53 ± 0.16), Classification Accuracy (93.5 ± 3.0%), TPR (68.7 ± 7.6%), FPR (4.3 ± 1.8%)	SD-based automated artifact detection and removal
Chang et al. (2016) [73]	Ocular (Eye blinks)	**R**: 24 subjects 10 trial × 15 s	Cognitive task	3 wet (Fp1, Fp2, vEOG)	vEOG	Classification Performance Metrics	TPR (∼99%), FPR (∼10%)	MSDW
Zhao et al. (2017) [74]	Ocular (S.N.S.); Muscular (S.N.S.)	**R**: 170 subjects 1 trial × 72/90 s	Resting-state; audio stimulation	3 n.r. (Fp1, Fp2, Fpz)	n.c.	Accuracy	Temporal trend comparison (numerical values n.r.)	Wavelet transform + Kalman filter
Thammasan et al. (2017) [75]	Ocular (Eye movements); Muscolar (S.N.S.); EMI (PLN)	**R**: 9 subjects 24 trials × 67/112 s	Resting-state; audio stimulation	8 soft dry (Fp1, Fp2, F3, F4, F7, F8, T7, T8)	Artifacts identified by visual inspection	Classification Performance Metrics on other topics	Enhancement on Classification Accuracy (n.r.), Enhancement on MCC (n.r.)	Automatic rejection based on Regression (pop_rejtrend), Joint Probability (pop_jointprob), Kurtosis (pop_rejkurt), FFT (pop_rejcont)
Hu et al. (2017) [76]	Ocular (Eye movements, blinks); Instrumental (EII, TEN)	**S**: n.r. × 8 s	n.a.	1 n.a. (n.a.)	Initial EEG	Classification Performance Metrics	Classification accuracy (95.8%)	Adaptive SSA
		**R**: 3 subjects 1 trial × 120 s	Resting-state	3 wet (frontal electrodes)	n.c.	Selectivity	Power spectrum differences (numerical values n.r.)	
Dehzangi et al. (2018) [77]	Ocular (Eye blinks)	**R**: 5 subjects 4 trials × 240/360 s	Cognitive task	7 wet (F7, Fz, F8, T7, T8, Pz, O2)	Artifact labels	Accuracy	DTW distances (multi-score detection performance: 87.4 ± 8.1%)	DTW score + K-means clustering + SVM
Cheng et al. (2019) [78]	Cardiac [79]; Ocular (Eye movements, blinks) [80,81]; Muscular (LiMC) [80,81]	**SS**: 11 subjects n.r. × 10 s	Resting-state; motor-imagery	n.r. wet (n.r.)	No contaminated EEG	Accuracy	RRMSE (triple contamination: SNR = 0.5: 0.23 ± 0.06; SNR = 1.0: 0.18 ± 0.04; SNR = 1.5: 0.15 ± 0.03)	SSA + ICA
						Selectivity	CC (triple contamination: SNR = 0.5: 0.78 ± 0.06; SNR = 1.0: 0.82 ± 0.04; SNR = 1.5: 0.85 ± 0.03)	
Val-Calvo et al. (2019) [82]	Ocular (Eye blinks)	**SS**: 15 subjects 15 trials × n.r.	Video stimulation	8 n.r. (AF3, T7, TP7, P7, AF4, T8, TP8, P8)	No contaminated EEG	Selectivity	CORR (0.87 (all bands), 0.86 (δ)), MI (0.66 (all bands), 0.64 (δ))	EAWICA
						Accuracy	RMSE (0.27 (all bands), 0.29 (δ))	
Grosselin et al. (2019) [83]	Ocular (Eye movements, blinks); Muscular (MaMC, TeMC); Instrumental (Clipping, electrode pop); Movement (Body)	**R1**: 3 subjects n.r.	Resting-state	32 wet (n.r.)	EEG baseline	Accuracy	SNR-based accuracy (SNR < 0 dB: 99.8%; 0 ≤ SNR < 10 dB: 82.5%; SNR ≥ 10 dB: 43.13%)	Classification-based approach
		**R2**: 21 subjects n.r.	Resting-state	2 dry (P3, P4)				
		**R3**: 10 subjects n.r. × 60 s	Resting-state (altert condition)	2 wet (P3, P4)		Classification Performance Metrics	Classification Accuracy (92.2 ± 2.2%)	
		**R4**: 10 subjects n.r. × 60 s	Resting-state (altert condition)	2 dry (P3, P4)				
Rosanne et al. (2019) [84]	Ocular (Eye blinks); Movement (Body, limb)	**R**: 48 subjects 6 trials × 1200 s	Dual-task	8 n.r. (FP1, FP2, AF7, AF8, T9, T10, P3, P4)	Raw EEG	Classification Performance Metrics on other topics	Enhancement on Classification Accuracy (no movement: (b) 10%; medium physical activity: (b) 4%; high physical activity: (a) 4%)	(a) ASR+wICA+ Random Forest, (b) ASR+ADJUST+ Random Forest
Inoue et al. (2019) [59]	Ocular (Eye movements, blinks); Musco- lar (OOcMC); Movement (Body)	**R**: 10 subjects 1 trial × n.r.	Resting-state; Motor task	8 n.r. (F3, C3, T3, O1, F4, C4, T4, O2)	Recorded video	n.c.	n.c.	Automatic detection algorithm based on frequency analysis
Blum et al. (2019) [85]	Ocular (Eye blinks)	**R**: 27 subjects 1 trial × n.r.	Resting-state; dual-task	24 wet (n.r.)	EEG baseline	Accuracy	SNR (numerical values n.r.)	Riemannian ASR
						Sensitivity	Blink amplitude (similarity value: 0.15)	
						Operational speed	Latency (5.6 ± 0.7 s)	
Butkevičiūtė et al. (2019) [86]	Movement (Body, limb)	**SS**: n.r 10 trials × 60 s	Motor tasks	n.r.	No contaminated EEG	Selectivity	Pearson’s correlation coefficient (0.055 ± 0.058)	BEADS + EMD
Albuquerque et al. (2019) [87]	Ocular (S.N.S.) Movement (Body, limb)	**R**: 47 subjects 2 trials × n.r.	Motor task	8 dry (T9, AF7, FP1, FP2, AF8, T10)	n.r.	Accuracy	PSD (ANOVA: 0.8715 ± 0.0699; mRMR: 0.8706 ± 0.0701), AMRC (ANOVA: 0.8815 ± 0.0521; mRMR: 0.8440 ± 0.0608)	wICA
Liu et al. (2019) [88]	Muscular (S.N.S.)	**SS**: 31 subjects n.r.	Resting-state	6 wet (n.r.)	No contaminated EEG	Accuracy	RRMSE (numerical values n.r.)	FMEMD-CCA
						Selectivity	CC (numerical values n.r.)	
Casadei et al. (2020) [89]	Other (generic large artifacts); Instrumental (Electrode pop)	**R**: 1 subject n.r.	n.r	1 n.r. (O2)	Band-pass filtered EEG	Selectivity	Amplitude and phase consistency (numerical values n.r.)	Model-based amplitude estimation
Islam et al. (2020) [90]	Instrumental (Cable movements); Movement (Body, limb)	**R**: 6 subjetcs, 9 trials × 240 s	Resting-state; motor tasks	21 dry (Fp1, Fp2, F7, F3, Fz, F4, F8, A1, T3, C3, Cz, C4, T4, A2, T5, P3, Pz, P4, T6, O1, O2)	EEG baseline	Accuracy	Artifact reduction rate (6.96 ± 2.96%), SNR (Δ SNR: 10.74 ± 4.24 dB), RMSE (Δ RMSE: 48.71 ± 36.14 mV)	Infomax ICA
						Selectivity	PSD distortion (improvement: 51.00 ± 21.36%), correlation (improvement: 77.31 ± 12.57%), coherence (improvement: 94.82 ± 5.54%)	
		**SS**: 5 subjects n.r.	Resting-state	n.r.	No contaminated EEG	Classification Performance Metrics	Classification Accuracy (90.8 ± 4.7%), TPR (84.4 ± 22.8%), FPR (45.1 ± 59.7%)	
Noorbasha et al. (2020) [91]	Ocular (S.N.S.)	**SS**: 3 subjects 1 trial × 120 s	Resting-state	3 wet (frontal electrodes)	No contaminated EEG	Accuracy	SNR-based RRMSE (SNR = 8 dB, RRMSE = 98%), MAE (Δ MAE: −17.43 ± 1.11 dB)	Ov-ASSA + ANC
Dey et al. (2020) [92]	Ocular (Eye blinks); Muscolar (CSMC)	**R**: 20 subjects 10 trials × 10 s	Resting-state	19 wet (n.r.)	n.r.	Classification Performance Metrics	Classification Accuracy (82.1 ± 2.9%), F1-score (0.800 ± 0.023)	MLP-based model
						Inference time	n.r.	
					n.a.	Hardware efficiency	Power consumption (over 70% reduction of model size with >3% loss in accuracy)	
Liu et al. (2021) [93]	Muscular (MaMC, TeMC)	**R**: 10 subjects 24 trials × 7 s	SSVEP	8 wet (POz, PO3, PO4, PO5, PO6, Oz, O1, O2)	EMG reference	Classification Performance Metrics	Classification Accuracy improvement (1-channel 24.42%, 3-channels 15.72%)	RLS Adaptive Filter
Kumaravel et al. (2021) [94]	Ocular (Eye blinks); Move- ment (Head, body, limb)	**R**: 6 subjects 3 trials × 25 s	SSVEP	8 wet (n.r.)	EEG baseline	Selectivity	SSVEP analysis (FTR improvement: 2 Hz–Correction 18.7%, 4 Hz–Removal 67.5%, 8 Hz–Removal 49.5%)	ASR
Shahbakhti et al. (2021) [95]	Ocular (Eye blinks)	**SS**: 1368 trials × 4104 s	n.a.	1 n.a. (n.a.)	Initial EEG	Classification Performance Metrics	TPR (95.77 ± 4.14%), FPR (0.0057 ± 0.007)	VME + DWT
						Accuracy	RRMSE (0.135 ± 0.031)	
						Selectivity	CC (0.955 ± 0.024), PSD difference (γ: 1.90±1.01, β: 6.02±2.73, α: 1.39±0.81, θ: 3.98±1.71, δ: 4.58±1.93)	
		**R**: 32 subjects 3000 trials × 9000 s	Motor-imagery; attention task	1 wet (frontal electrode)	Raw EEG	Classification Performance Metrics	TPR (95.3 ± 2.3%), FPR (0.0074 ± 0.0024)	
Sha’bani et al. (2021) [96]	Ocular (Eye blinks)	**SS**: 36 subjects n.r. × 1280 s	Resting-state	n.r. (focus on AF3)	No contaminated EEG	Accuracy	RMSE (7.62 ± 2.51)	EEMD + OD + cubic spline interpolation
						Selectivity	Pearson’s correlation (0.802 ± 0.102), PDS differences (δ: 7.11 ± 2.90; θ: 1.68 ± 0.79; α: 1.99 ± 1.41; β: 10.09 ± 13.29; γ: 7.80 ± 9.77), SAR (∼12)	
Aung et al. (2021) [97]	Instrumental (Cable movements)	**R**: 6 subjects 24 trials × 540 s	Resting-state	2 n.r. (Fpz e Fp1)	No contaminated channel	Classification Performance Metrics	Classification Accuracy (86.2 ± 5.9%), TPR (84.8 ± 6.3%), FPR (2.0 ± 4.5%)	M-mDistEn
Zhang et al. (2021) [58]	Ocular (Eye blinks); Muscular (MaMC, TeMC, PMC, LaMC, ToMc, NMC); Movement (Tremor); EMI (PLN, S.N.S.)	**SS**: n.r. n.r. × 10 s	n.a.	1 n.a. (n.a.)	Initial EEG	Accuracy	RMSE (Non-Blink zones: 0.59 ± 0.07; Blink zones: 2.81 ± 0.38)	DWT + CCA
						Selectivity	CC (Non-Blink zones: 0.947 ± 0.003; Blink zones: 0.167 ± 0.027)	
		**R**: 23 subjects 1 trial × n.r.	Sleep	23 n.r. (focus on C4, P7, FT9, FP1)	Raw EEG	Selectivity	CC (0.923 ± 0.048), MI (1.00 ± 0.33), MSC plot (numerical values n.r.)	
Noorbasha et al. (2021) [98]	Instrumental (Cable movements) [79]	**R**: 6 subjects 4 trials × 540 s	Resting-state	2 n.r. (P2, P1)	No contaminated EEG	Accuracy	SNR (Δ SNR: 1.6 dB (0.79% overlap)), RRMSE (improvement rate: 15.62% (0.79% overlap))	SSA with modified grouping
						Operational speed	Latency (0.84 s ((0.79% overlap))	
Ingolfsson et al. (2022) [99]	Ocular (Eye movements), Instru- mental (Electrode pop, displace- ment); Muscular (MaMC, TeMC); Movement (Tremor)	**R**: 213 subjects n.r.	n.r.	22 n.r. (focus on F7, T3, T3, T5, F8, T4, T4, T6)	Artifact labels	Classification Performance Metrics	Classification Accuracy (87.8 ± 1.5%), F1-score (0.850 ± 0.019)	DWT + MMC
Chen et al. (2022) [100]	Ocular (Eye movements, blinks); Musco- lar (MaMC, TeMC, CSMC); Movement (Head); EMI (PLN)	**R**: 32 subjects 6 trials × 720 s	Audio and video stimulation	8 wet (F3, F4, C3 C4, T3, T4, O1, O2)	Raw EEG	Selectivity	PSD differences (numerical values n.r.)	MRA + CCA + SVM OD
		**SS**: 32 subjects n.r. × 1200 s	n.r.	8 wet (F3, F4, C3 C4, T3, T4, O1, O2)	No contaminated EEG	Accuracy	NSR-based RRMSE (NSR = 6 dB, Ocular: 12.7 ± 2.2; Muscular and Movement: 14.2 ± 2.5; PLN continous 10.3 ± 1.6; PLN intermittent 12.0 ± 1.9)	
		**R**: 12 subjects 440 trials × 880 s	Video stimulation	3 wet (Cz, Pz, Oz)	Artifact-related ICs	Selectivity	ERP peak amplitudes (numerical values n.r.)	
Occhipinti et al. (2022) [101]	Muscular (MaMC, TeMC, PMC, LaMC, ToMC) Movement (Body)	**R**: 12 subjects 1 trial × 120 s	Resting-state; cognitive tasks	1 wet (into the ear canal)	Raw EEG	Selectivity	Amplitude and mean power reduction rate (numerical (value n.r.))	NA-MEMD
Paissan et al. (2022) [102]	Ocular (Eye movements, blinks) [103]; Muscular (CSMC, ZMC, OOrMC, OOcMC, MaMC) [103]	**SS**: 105 subjects 1 trial × n.r.	Resting-state; motor tasks	n.r.	No contaminated channel	Classification Performance Metrics	SNR-based classification accuracy (SNR = 3 dB) (classification accuracy = 75%)	1D-CNN with HPO
Peh et al. (2022) [104]	Ocular (Eye movements); Muscular (MaMC, TeMC, S.N.S.); Instrumen- tal (Electrode pop); Movement (Tremor)	**R**: 310 subjects 1 trial × n.r.	Resting-state; dual-task	19 n.r. (Fp1, F3, C3, P3, F7, T3, T5, O1, Fz, Cz, Pz, Fp2, F4, C4, P4, F8, T4, T6, O2)	Artifact labels	Classification Performance Metrics	Balanced Accuracy (muscolar: 0.95); (intrumental: 0.73); (ocular: 0.83); (movement: 0.86), TPR (49.2 ± 10.3%), FPR (3.0 ± 1.6%)	CNN with BM loss
Brophy et al. (2022) [105]	Ocular (S.N.S.); Muscular (S.N.S.); EMI (PLN)	**SS**: n.r.	Motor-imagery	n.r.	No contaminated EEG	Accuracy	RRMSE (numerical values n.r.)	GAN
						Selectivity	CC (numerical values n.r.), PSD differences (numerical values n.r.)	
Xiao et al. (2022) [106]	Cardiac; Ocular (Eye movements); Muscular (s.n.s); Instrumental (EII); EMI (PLN)	**R**: 28 subjects 40 trials × 20–60 s	Resting-state	22 wet (n.r.)	Raw EEG	Selectivity	Spectrum differences numerical values n.r.)	Modified ASR method based on spectral properties
					n.a.	Hardware efficiency	Power consumption (numerical values n.r.)	
Arpaia et al. (2022) [107]	Ocular (Eye mo- vements, blinks); Muscular (S.N.S.)	**R**: 13 subjects 1 trial × 900–2700 s	Resting-state	27 n.r.	EEG baseline	Accuracy	RMSE (numerical values n.r.)	ASR
						Selectivity	SD differences (numerical values n.r.)	
Noorbasha et al. (2022) [108]	Instrumental (Electrode pop, EII)	**R**: n.r. 5 trials × 5 s	Resting-state	18 n.r.	Raw EEG	Accuracy	MAE (0.0282 ± 0.0211)	SWT + GSTV
						Selectivity	PSD differences (numerical values n.r.)	
		**SS**: 22 trials × 5 s	n.a.	1 n.a. (n.a.)	Initial EEG	Accuracy	RRMSE (SNR = 6 dB, 0.45 ± 0.05)	
						Selectivity	CC (SNR = 6 dB, 0.86 ± 0.03)	
Zhang et al. (2022) [109]	Cardiac; Ocular (Eye movements, Blinks) Muscular (S.N.S.) Instrumental (Electrode displacement)	**SS**: 27 subjects 2 trials × 30 s	Resting-state	19 n.r. (Fp1, Fp2, F3, F4, C3, C4, P3, P4, 01, 02, F7, F8, T3, T4, T5, T6, Fz, Cz, Pz)	No contaminated EEG	Accuracy	RRMSE (mixed artifacts: 0.60)	GRU-MARSC
						Selectivity	CC (mixed artifacts: 0.81), PSD differences (numerical values n.r.)	
						Classification Performance Metrics	classification accuracy (98.52%), PPV (98.22%), TPR (98.81%)	
						Operational speed	Latency (10,250 samples, 11.05 s)	
Jayas et al. (2023) [110]	Ocular (Eye movements, blinks) Muscular (S.N.S.)	**R**: 6 subjects n.r.	Motor task	8 wet (4 in each ear, 2 in front and back of the ear, 2 in upper and bottom)	Co-registered scalp-EEG	Accuracy	RMS (n.r.), SNR (n.r.), ZCR (n.r.), Max Gradient (n.r.)	Classification model based on Random Forest
						Selectivity	Skewness (n.r.), Kurtosis (n.r.), Spectral Entropy (n.r.), ACF (n.r.)	
						Classification Performance Metrics	Classification accuracy (76.70%) F1-score (0.85%)	
Narmada et al. (2023) [111]	Cardiac; Ocular (Eye movements); Muscular (LiMC)	**SS**: (a) 22 subjects n.r. × 8 s; (b) 9 subjects 576 trials × 8 s	(a) n.r.; (b) Motor-imagery	(a) n.r.; (b) 22 n.r.	No contaminated EEG	Accuracy	MAE (Cardiac: 1.13 ± 0.64; Muscular: 1.22 ± 1.18; Ocular: 0.81 ± 0.19), PSNR (Cardiac: 44.76 ± 2.13; Muscular: 44.74 ± 3.88; Ocular: 46.05 ± 0.95), RMS (Cardiac: 1.17 ± 0.64; Muscular: 1.29 ± 1.20; Ocular: 0.85 ± 0.19)	Deep learning + adaptive wavelet
						Selectivity	CC (Cardiac: 1.174 ± 0.006; Muscular: 1.137 ± 0.036; Ocular: 1.177 ± 0.0003)	
						Efficiency	CSED (Cardiac: 1373.9 ± 0.65; Muscular: 1374.8 ± 5.16; Ocular: 1378.8 ± 2.68)	
Mahmud et al. (2023) [112]	Instrumental (Cable Movement) [64]	**R**: 6 subjects 4 trials × 540 s	Resting-state	2 n.r. (Fpz, Fp1h)	No contaminated channel	Accuracy	DSNR (26.641 dB), MAE (0.056 ± 0.025), artifact reduction rate (90.52%)	Deep learning + adaptive wavelet
						Selectivity	PSD comparison (numerical values n.r.)	
Jiang et al. (2023) [113]	Ocular (Eye blinks)	**SS**: 27 subjects 1 trial × n.r.	Resting-state	1 dry (Fp1)	No contaminated EEG	Classification Performance Metrics	TPR (92.86%), FPM (0.85)	VME+ MFE + GWO
		**R**: 9 subjects n.r. × 480–900 s	Resting-state	1 n.r. (Fp1/Fp2)	Expert-annotated blinks	Classification Performance Metrics	CCR (97.63%), TPR (92.64%), FPM (0.02), FDR (2.37%)	
Cui et al. (2023) [60]	Cardiac [79]; Ocular (Eye movements, blinks) [103]; Muscular (CSMC, ZMC, OOrMC, OOcMC, MaMC) [103]; Movement (Body) [64]	**SS**: 158 subjects 1 trial × n.r.	Resting-state	1 n.a. (n.a.)	No contaminated EEG	Accuracy	RRMSE (Muscular: 0.356; Ocular: 0.210; Cardiac: 0.273; Movement: 0.262), SNR (Muscular: 9.463; Ocular: 14.653; Cardiac: 10.275; Movement: 11.951)	EEGIFNet
						Selectivity	CC (Muscular: 0.926; Ocular: 0.974; Cardiac: 0.951; Movement: 0.945)	
						Efficiency	CSED (flop of 100.784 M)	
Kaongoen et al. (2023) [61]	Ocular (Eye movements, blinks); Instrumental (Cable movements)	**SS**: (i) 24 subjects 1 trial × 540 s [79] (ii) 33 subjects n.r. [114]	Resting-state	1 n.a. (n.a.)	No contaminated EEG	Accuracy (n.r.)	MSE (Δ MSE: 9.39 ± 1.45), SNR (Δ SNR: 15.24 ± 0.52)	WT + ASR
						Selectivity (n.r.)	CC (0.210 ± 0.095)	
Kumaravel et al. (2023) [115]	n.c.	**R1**: 6 subjects 3 trials × 25 s	SSVEP	8 dry (n.r)	Raw EEG	Selectivity	FTR (2 Hz: 1.3 ± 0.3; 4 Hz: 3.0 ± 0.8; 8 Hz: 5.5 ± 1.5)	IMU-ASR
		**R2**: 37 subjects 4 trials × 600 s	Motor task	120 wet (n.r.)	Raw EEG	Selectivity	Brain ICs (No.: 6 ± 3), muscle ICs (No.: 12 ± 9)	
Li et al. (2023) [116]	Ocular (Eye movements, blinks); Muscular (CSMC, ZMC, OOrMC, OOcMC, MaMC); Movement (S.N.S.)	**SS**: (i) 27 subjects, 2 trials × n.r. [114] (ii) 105 subjects, 1 trial × n.r. [103]	(i) Resting-state (ii) Resting-state, motor tasks	(i) 19 n.r. (FP1, FP2, F3, F4, C3, C4, P3, P4, O1, O2, F7, F8, T3, T4, T5, T6, Fz, Cz, Pz); (ii) n.r.	Raw EEG	Accuracy	RRMSE (0.4129 ± 0.0979), SNR (6.0321 ± 1.8962)	ResUnet1D-RNN
						Selectivity	CC (90.75% ± 4.27)	
		**R [117]**: 23 subjects, 23–26 trials × n.r.	Resting-state	n.r.	Raw EEG	Selectivity	Waveform and PSD qualitative analysis (numerical values n.r.)	
Yin et al. (2023) [118]	Ocular (Eye movements, blinks)	**SS**: 27 subjects from [114] n.r.	Resting-state	8 n.r. (FP1, FP2, F3, F4, F7, F8, T3, T4)	No contaminated EEG	Accuracy	SNR (11.123 ± 1.306), RRMSE (0.340 ± 0.044)	GCTNet Generator (CNN and Transformer Blocks)
						Selectivity	CC (0.929 ± 0.015)	
O’Sullivan et al. (2023) [119]	Instrumental (Poor electrode contact); Muscular (S.N.S.): Movement (S.N.S.); Cardiac	**R**: 51 subjects n.r.	Daily activities	9 disposable (F3, F4, C3, C4, Cz, T3, T4, O1, O2)	Raw EEG	Classification Performance Metrics	AUC (0.844), MCC (0.649), Classification Sensitivity (0.794), Classification Specificity (0.894)	CNN deep learning architecture
Chen et al. (2023) [120]	Ocular (Eye movements, blinks); Muscular (Head movement)	**SS**: 52 subjects from [103] n.r.	Motor-imagery	1 n.r. (n.r.)	No contaminated EEG	Accuracy	RRMSE (0.444)	Denoiseformer Tranfomer-based Encoder and Self-Attentional Mechanism
						Selectivity	CC (0.859)	
Hermans et al. (2023) [121]	Instrumental (Device interference, electrodes); Muscular (S.N.S.): Movement (S.N.S.); Cardiac	**R**: 133 subjects n.r.	Daily Activities	8 n.r. (Fp1, Fp2, C3, C4, T3, T4, O1, O2)	Raw EEG	Classification Performance Metrics	Classification Accuracy (96.6%), F1-score (86.2), Miss Rate (11.7)	Semi-supervised multi-task CNN (encoder + decoder)
Bahadur et al. (2024) [122]	Ocular (Eye movements, blinks)	**SS**: 27 subjects n.r.	Resting-state	19 n.r.	No contaminated EEG	Accuracy	RMSE (2.22 ± 0.27)	DWT + LMM
						Selectivity	CC (0.93 ± 0.02)	
					n.a.	Hardware efficiency	Silicon area (area: 5181.73 μm^2^, power: 446.06 μW)	
Arpaia et al. (2024) [123]	Ocular (Eye mo- vements, blinks); Muscular (TeMC, MaMC); Movement (Head)	**R**: 2 subjects 50 trials × 40 s	Resting-state	8 dry (Fz, C3, Cz, C4, Pz, PO7, PO8, Oz)	EEG baseline	Accuracy	RRMSE (4-channels (k = 9): contaminated 5.0 ± 0.0, clean 4.5 ± 2.0; 3-channels (k = 8): contaminated 6.0 ± 4.0, clean 5.5 ± 3.5; 2-channels (k = 9): contaminated 3.0 ± 0.0, clean 3.0 ± 0.0)	MEMD + ASR
Ingolfsson et al. (2024) [124]	Ocular (Eye mo- vements); Musco- lar (MaMC, TeMC, S.N.S.) Movement (Tremor)	**R**: 22 subjects n.r.	Resting-state	4 n.r. (F7-T7, T7-P7, F8-T8, T8-P8)	Artifact labels	Classification Performance Metrics	Classification Accuracy (93.95%), Sensitivity (61.27 ± 5.66%), FPR (FP-h) (<0.58)	XGBoost-based model
Saleh et al. (2024) [125]	Ocular (Eye mo- vements) [114]; Muscular (CSMC, ZMC, OOrMC, OOcMC, MaMC) [103]; Instrumental (Electrode displacement) [64]	**SS**: 138 subjects 1 trial × n.r.	Resting-state; motor tasks	64 n.r. (n.r.)	No contaminated EEG	Accuracy (n.r.)	RRMSEt (Ocular: 0.52 ± 0.10; Movement: 0.70 ± 0.10; Muscular: 0.58 ± 0.16; Clean EEG: 0.30 ± 0.07) RRMSE_*f*_ (Ocular: 0.53 ± 0.20; Movement: 0.72 ± 0.17; Muscular: 0.59 ± 0.18; Clean EEG: 0.42 ± 0.11)	Deep Convolutional Autoencoder
						Selectivity	CC (Ocular: 0.86 ± 0.06; Movement: 0.71 ± 0.11; Muscular: 0.80 ± 0.12; Clean EEG: 0.95 ± 0.02)	
Nair et al. (2025) [126]	Ocular (Eye movements); Muscular (MeMC, SubMc)	**R**: 197 subjects 2 trials × 72,000 s	Resting-state	2 n.r. (n.r.)	Raw EEG	Accuracy (n.r.)	RMSE (3.0 ± 0.5 μV), SNR (22.5 ± 1.2 dB)	CNN + LMS (OBC Radix-4 DA)
						Selectivity	CC (0.93 ± 0.02)	

**Table 2:** Details of public datasets considered in the included studies. The datasets are defined by data type (R = real, S = simulated, SS = semi-simulated), signal category (physiological or artifact), and experimental protocol, including environmental conditions, subject position, and stimulus/task. For each dataset, the table reports participants’ information (number and type, age (mean ± SD), sex (Male (M), Female (F))), hardware setup (device, number and type of electrodes), and signal processing stage. “Not Applicable” (n.a.) indicates parameters not relevant in the context (e.g., acquisition setup for simulated data). “Not Reported” (n.r.) refers to parameters relevant but unspecified by the authors (e.g., participant sex). “Not Considered” (n.c.) refers to parameters relevant but not used in the study (e.g., absence of pre-processing). An explanatory table for other technical acronyms is recommended at the end of the document.

Public Dataset	Data Type	Signal Category	Experimental Protocol	Participants	Hardware	Signal Processing
EEG datasets for motor-imagery brain–computer interface [133]	R	Reference (Physiological EEG and Ocular and EMG Artifact)	**Ambiental Conditions**: laboratory setting; background noise level was held between 37 and 39 decibels **Subject Position**: sitting in a chair with armrests in front of a monitor **Stimulus/Task**: (i) motor-imagery movement of left and right hands (ii) resting state EO and (iii) artifact recordings (eye blinking, eyeball movement up/down and left/right, head movement, and jaw clenching)	**No. and Type**: 52 healthy subjects **Age**: 24.8 ± 3.9 years **Sex**: 19 F, 33 M	**Device**: Biosemi ActiveTwo system **Electrodes No.**: 64 **Electrodes Type**: n.r.	Common average reference, fourth order Butterworth filter [8–30 Hz]
BCI Competition 2008 Graz data set B [80]	R	Reference (Physiological EEG and EOG Artifact)	**Ambiental Conditions**: n.r. **Subject Position**: sitting in an armchair in front of an LCD computer monitor placed approximately 1 m in front at eye level **Stimulus/Task**: (i) motor-imagery movement of the left and right hands, (ii) rest EO (while looking at a fixation cross on the screen) (iii), rest EC (iv), eye movements (eye blinking, rolling, up–down, left–right movements)	**No. and Type**: 10 healthy subjects **Age**: 24.7 ± 3.3 years **Sex**: 6 M and 4 F	**Device**: Easycap **Electrodes No.**: 3 bipolar recordings extracted from the 22 total electrodes. EOG was recorded with 3 monopolar electrodes. **Electrodes Type**: n.r.	BPF [0.5–100 Hz] and notch filter at 50 Hz
BCI Competition 2008 Graz data set A [81]	R	Reference (Physiological EEG and EOG Artifact)	**Ambiental Conditions**: n.r. **Subject Position**: sitting in a comfortable armchair in front of a computer screen **Stimulus/Task**: (i) four motor-imagery tasks (left and right hand, both feet, and tongue) (ii) EO (looking at a fixation cross on the screen), (iii) EC, (iv) eye movements	**No. and Type**: 9 healthy subjects **Age**: n.r. **Sex**: n.r.	**Device**: n.r. **Electrodes No.**: n.r. **Electrodes Type**: n.r.	n.r.
Ear-EEG Recording for Brain Computer Interface of Motor Task [134]	R	Reference (Physiological EEG)	**Ambiental Conditions**: n.r. **Subject Position**: sitting in front of a computer monitor **Stimulus/Task**: subjects were asked to imagine and grasp the left or right hand according to an arrow direction present on the computer monitor	**No. and Type**: 6 healthy subjects **Age**: 22–28 years **Sex**: 2 M, 4 F	**Device**: Neuroscan Quick Cap (Model C190) **Electrodes No.**: n.r. for the cap-EEG. Ear-EEG were recorded with 8 ear electrodes **Electrodes Type**: n.r.	BPF [0.5–100 Hz] together with a notch filter
A Methodology for Validating Artifact Removal Techniques for Physiological Signals [64]	R	Reference (Physiological EEG and Cable Motion Artifact)	**Ambiental Conditions**: n.r. **Subject Position**: n.r. **Stimulus/Task**: subjects were asked to keep their eyes closed and maintain a stationary head position throughout the experiment. An artifact motion was then induced to one of the electrodes by pulling on the connecting lead.	**No. and Type**: 6 healthy subjects **Age**: 27.0 ± 4.3 years **Sex**: 3 M, 3 F	**Device**: Electro-cap International **Electrodes No.**: 2 electrodes were considered based on the 256 electrodes composing the EEG device **Electrodes Type**: wet	n.r.
The impact of the MIT-BIH Arrhythmia Database [79]	R	Reference (Physiological ECG)	**Ambiental Conditions**: clinical setting inside the Boston’s Beth Israel Hospital (BIH; now the Beth Israel Deaconess Medical Center) **Subject Position**: n.r. **Stimulus/Task**: n.r.	**No. and Type**: 23 healthy subjects and 24 participants with uncommon but clinically important arrhythmia (47 subjects in total) **Age**: 23 to 89 years **Sex**: 25 M, 22 F	**Device**: n.r. **Electrodes No.**: n.r. **Electrodes Type**: n.r.	n.r.
TUH EEG Artifact Corpus dataset [135]	R	Reference (Physiological EEG)	**Ambiental Conditions**: data are composed of archival records acquired in clinical settings in the Temple University Hospital (TUH) **Subject Position**: n.r. **Stimulus/Task**: n.r.	**No. and Type**: archival recordings of 10.874 healthy and clinical subjects **Age**: 1 to 90 years **Sex**: 51% F, 49% M	**Device**: n.r. **Electrodes No.**: n.r. **Electrodes Type**: n.r.	n.r.
SEED database [136]	R	Reference (Physiological EEG and EOG Artifact)	**Ambiental Conditions**: n.r. **Subject Position**: n.r. **Stimulus/Task**: subjects were asked to watch 15 film clips designed to elicit positive, neutral, and negative emotions	**No. and Type**: 15 healthy subjects **Age**: 23.3 ± 2.4 years **Sex**: 7 M, 8 F	**Device**: ESI NeuroScan System for EEG signals acquisition and SMI eye-tracking glasses for eye movements **Electrodes No.**: n.r. **Electrodes Type**: n.r.	BPF [0–75 Hz] was applied
CHB-MIT Scalp EEG Database [117]	R	Reference (Physiological EEG)	**Ambiental Conditions**: n.r. **Subject Position**: n.r. **Stimulus/Task**: EEG recordings were acquired during and after seizures attacks	**No. and Type**: 22 pediatric subjects with intractable seizures **Age**: 1.5 to 22 years **Sex**: 5 M, 17 F	**Device**: n.r. **Electrodes No.**: n.r. **Electrodes Type**: n.r.	n.r.
Freiburg EEG dataset [137]	R	Reference (Physiological EEG)	**Ambiental Conditions**: clinical settings in the Epilepsy Center of the University Hospital of Freiburg, Germany **Subject Position**: n.r. **Stimulus/Task**: EEG recordings were made during an invasive pre-surgical epilepsy monitoring	**No. and Type**: 21 patients suffering from medically intractable focal epilepsy **Age**: n.r. **Sex**: n.r.	**Device**: Neurofile NT digital video EEG system **Electrodes No.**: 128 depth-electrodes **Electrodes Type**: n.r.	No notch or BPF have been applied
A large EEG motor-imagery dataset for EEG brain computer interfaces [138]	R	Reference (Physiological EEG)	**Ambiental Conditions**: n.r. **Subject Position**: comfortably sitting in a recliner chair in front of a computer screen positioned approximately 200 cm in front at slightly above the eye level **Stimulus/Task**: (i) three motor imageries of left and right-hand movements and one passive mental imagery in which participants remained neutral and engaged in no motor imagery, (ii) imagery of left and right leg movement and tongue movements, (iii) imaginary movements of the fingers on one hand, (iv) to remain passive throughout the experiment.	**No. and Type**: 13 healthy subjects **Age**: 20 to 35 years **Sex**: 8 M, 5 F	**Device**: EEG-1200 JE-921A EEG system (NihonKohden, Japan) with the help of an Electro-Cap International **No. of Electrodes**: 19 **Electrodes Type**: wet	BPF [0.53–70 Hz] and a 50 Hz notch filter
Prediction of Reaction Time and Vigilance Variability From Spatio-Spectral Features of Resting-State EEG in a Long Sustained Attention Task [139]	R	Reference (Physiological EEG)	**Ambiental Conditions**: in a dimly lit EEG room within a Faraday cage, in the early afternoon **Subject Position**: comfortably sitting in a chair 20 cm away from a 17-inch LCD monitor **Stimulus/Task**: (i) resting session with EO, (ii) resting-state with EC, (iii) SART sessions: pressing the left mouse button when any digit appeared on the screen except for the digit 3, in which case responses should be withheld	**No. and Type**: 10 healthy subjects **Age**: 30.3 ± 6.9 years **Sex**: 6 F, 4 M	**Device**: n.r. **Electrodes No.**: 64 **Electrodes Type**: n.r.	n.r.
Impact of Stimulus Features on the Performance of a Gaze-Independent Brain-Computer Interface Based on Covert Spatial Attention Shifts [140]	R	Reference (Physiological EEG and EOG Artifact)	**Ambiental Conditions**: in an acoustically shielded and dimly lit cabin **Subject Position**: sitting in front of a 24” display from a distance of 70 cm **Stimulus/Task**: (i) horizontally and vertically track with their gaze a cross presented on the display or blink when the cross was replaced by a circle, (ii) respond to yes/no questions or statements by shifting their attention to a green-cross to respond with “yes” or to a red-cross to respond with “no”.	**No. and Type**: 18 healthy participants **Age**: 19 to 38 years **Sex**: 10 F, 8 M	**Device**: n.r. **Electrodes No.**: 29. Furthermore, vertical and horizontal EOG were simultaneously recorded. **Electrodes Type**: n.r.	n.r.
A semi-simulated EEG/EOG dataset for the comparison of EOG artifact rejection techniques [114]	R	Reference (Physiological EEG)	**Ambiental Conditions**: n.r. **Subject Position**: n.r. **Stimulus/Task**: (i) EC condition, (ii) EO condition	**No. and Type**: 27 healthy subjects **Age**: 28.2 ± 7.5 years for M participants, 27.1 ± 5.2 years for F participants **Sex**: 14 M, 13 F	**Device**: n.r. **Electrodes No.**: 19 for cap EEG; 4 for EOG **Electrodes Type**: n.r.	BPF [0.5–40 Hz] for cap EEG and [0.5–5 Hz] for EOG with a notch filter at 50 Hz. Obtained data were then inspected to ensure no significant contamination by external artifacts.
A Survey on the Feasibility of Surface EMG in Facial Pacing [141]	R	Reference (EMG Artifact)	**Ambiental Conditions**: n.r. **Subject Position**: n.r. **Stimulus/Task**: (i) voluntary smile, lip pucker, and frown movement tasks, (ii) smile and pucker movement while chewing gum	**No. and Type**: 12 healthy subjects **Age**: 31 to 55 years **Sex**: 6 F, 6 M	**Device**: NeXus-10 physiological monitoring device (Mind Media BV) **Electrodes No.**: n.r. **Electrodes Type**: pre-gelled	8th order Butterworth filters implemented as zero-phase forward and reverse ones used to remove 50 Hz power line noise and to limit the signal frequencies to the range of 20–500 Hz.
Assessing the effects of voluntary and involuntary eyeblinks in independent components of EEG [142]	R	Reference (Physiological EEG and EOG Artifact)	**Ambiental Conditions**: in a dim room (mean illuminance: 188.95 ± 24.50 lx) **Subject Position**: the distance between subject and display was roughly 60 cm **Stimulus/Task**: (i) focus on a black cross-fixation in the center of the display and to blink with both eyes after a sound stimulus, (ii) press the key corresponding to the associated sound after a sound stimulus	**No. and Type**: 20 healthy subjects **Age**: 22.8 ± 1.5 years **Sex**: 14 M, 6 F	**Device**: n.r. **Electrodes No.**: 14 for cap EEG and 2 for EOG **Electrodes Type**: wet	Butterworth BPF [0.5–60 Hz]
EEGdenoiseNet [103]	R	Reference (Physiological EEG and Ocular and EMG Artifact)	**Ambiental Conditions**: laboratory setting; background noise level was held between 37 and 39 decibels **Subject Position**: sitting in a chair with armrests in front of a monitor **Stimulus/Task**: (i) motor-imagery movement of left and right hands, (ii) resting state EO, and (iii) artifact recordings (eye blinking, eyeball movement up/down and left/right, head movement, and jaw clenching)	**No. and Type**: 52 healthy subjects **Age**: 24.8 ± 3.9 years **Sex**: 19 F, 33 M	**Device**: Biosemi ActiveTwo system **Electrodes No.**: 64 **Electrodes Type**: n.r.	Physiological data were preprocessed with BPF [1–80 Hz] and a 50 Hz notch filter. Then, the not-brain components were removed after ICA. Finally, a visual inspection by an expert validated the clean procedure.

**Table 3:** Parameters of artifact detection pipelines proposed in the included studies. R = real recordings; S = simulated signals; SS = semi-simulated signals. “Not Applicable” (n.a.) indicates parameters not relevant in the context (e.g., acquisition setup for simulated data). “Not Reported” (n.r.) refers to parameters relevant but unspecified by the authors (e.g., participant sex). “Not Considered” (n.c.) refers to parameters relevant but not used in the study (e.g., absence of pre-processing). An explanatory table for other technical acronyms is recommended at the end of the document.

Article	Pre-Processing	Epoching	Feature Extraction				Feature Selection	Classification/ Decision Rule	Channel Specificity
			Domain	Method	Feature	Hyperparameters			
Sweeney et al. (2012) [64]	n.r.	n.r.	Time	Adaptive Filter	Artifact estimate time series	optimization method = NLMS, reference signal = triaxial accelerometer, length = n.r.	n.c.	Manual detection based on shape, frequency, and amplitude	Single-channel
				Kalman Filter	Predictor estimate time series	n.r.			
				EEMD-ICA	ICs	EEMD: ensemble = 100, noise = n.r., contrast function = n.r.; ICA: n.r.;			
Matiko et al. (2013) [65]	n.r.	1 s; overlap n.c.	Frequency	MCA (using STFT)	Basis coefficients	MCA: number of components = 2; STFT: window length = 500 ms (for eye blinks), 2 s (for EEG);	n.c.	n.c. (clean signal reconstruction)	Single-channel
Peng et al. (2013) [66]	**S/R**: BPF (0.5–40 Hz)	40 s; overlap n.r.	Time–frequency	DWT	Wavelet coefficients	mother wavelet = Daubechies 4, decomposition layers = 7	n.c.	Thresholding	Single-channel
Mihajlovic et al. (2014) [67]	**R**: 3rd order Butterworth SBF (49–51 Hz)	2 s; 75% overlap	Frequency	Welch method	PSD and coherence	optimization method = LLMS, forgetting factor = 0.1, step size = 0.5, reference signal = ETI	n.c.	n.c.	Single-channel
Zhao et al. (2014) [68]	**R**: BPF (0.5–45 Hz) **S**: n.c.	n.r.	Time–frequency	DWT	Wavelet coefficients (edge)	mother wavelet = Daubechies 7, decomposition layers = 5; mother wavelet = Haar, decompo- sition layers = 5	n.r.	Thresholding	n.r.
Majmudar et al. (2015) [69]	n.r.	0.5 s; ∼31% overlap	Time	Algebraic approach FIR-based	Mean	n.r.	n.c.	Thresholding	Single-channel
D’Rozario et al. (2015) [72]	**R**: HPF (0.3 Hz), LPF (35 Hz), Low BPF (100 Hz), Notch (50 Hz); **S**: n.c.	5 s; no overlap	Time	SD-based automatic artifact detection	SD	n.r.	n.c.	Thresholding	Multi-channel
Kim et al. (2015) [70]	n.c.	n.r.	Time	FastICA	ICs	No. of ICs = 10	n.c.	SVM	Multi-channel
Rahman et al. (2016) [71]	n.c.	n.r.	Time	SG filter	n.r.	n.r.	n.c.	n.r.	Single-channel
Chang et al. (2016) [73]	**R**: 1st order Butterworth HPF (0.1 Hz), Downsampling (64 Hz), Median filter	n.r.	Time	SDW, MSDW	LMM	window size = same as the half-width of the artifact, determined by an empirical procedure	n.c.	Thresholding	Single-channel
Zhao et al. (2017) [74]	**R**: Mid-filter, FIR filter (1–40 Hz)	2 s; overlap n.r.	Frequency	Welch method	n.r.	n.r.	n.r.	Thresholding	Single-channel
			Time–frequency	Wavelet	n.r.	n.r.			
Thammasan et al. (2017) [75]	**R**: HPF (1 Hz), Notch (60 Hz ± 1 Hz bandwidth)	4 s, 3.5 s; overlap n.r.	Time	Regression	Trend Slope	threshold cutoff = 0.3	n.c.	Thresholding	Multi-channel
				Joint Probability	SD	threshold cutoff = 3*SD			
				n.r.	Kurtosis	n.r.			
			Frequency	FFT	multi-taper PSD	taper bandwidth = 5 Hz, sliding window = 1 s, number of tapers = 9			
Hu et al. (2017) [76]	n.r.	n.r.	Time	Adaptive SS	RCs	window length = 40	n.c.	Thresholding	Single-channel
Dehzangi et al. (2018) [77]	n.r.	0.5 s; 90% overlap	Time	K-means clustering	DTW multi-score space positioning	K clusters = 2	n.c.	K-means clustering + SVM	n.r.
Cheng et al. (2019) [78]	**SS**: BPF (1–70 Hz) (for EEG); BPF (0.5–10 Hz) (for EOG)	n.r.	Time	SSA-ICA	Autocorrelation	SSA: window lenght = 130; ICA: No. of ICs = 13;	n.c.	Thresholding	Single-channel
Val-Calvo et al. (2019) [82]	**SS**: IIR Notch (50 Hz), BPF (1–50 Hz)	n.r.	Time + Frequency	EAWICA	WICs + Kurtosis and Renyi entropy	WAVELET: n.r.; ICA: No. of ICs = 8;	Chi-squared statistic-based feature selection	Thresholding	All channels
Grosselin et al. (2019) [83]	**R1**: DC offset removal, Notch (50 Hz) **R2/R3**: n.r.	**R1**: 1 s; overlap n.c. **R2/R3**: n.r.	Time	n.a.	Statistical features, EEG bands max value, SD, kurtosis, skewness	n.r.	FCBF	Weighted k-NN	Single-channel
			Frequency	FFT	SEF (80%, 90%,95%), Spectral Moments (0, 1st, 2nd), Power Spectrum Centre Frequency, Spectral RMS, ISD, SNR, Modified Median/ Mean Freq., Ratio Spectrum Area, Non-normalized Power, LogP, RP, Wavelet energy; Cospectral Coefficients, Frequency- filtered energies, RSD; Shannon entropy, spectral entropy, SVD entropy	n.r.			
Rosanne et al. (2019) [84]	**R**: FIR BPF (1–45 Hz)	8 s, 7 s; overlap n.r.	Time	ASR (PCA)	Short-window variance	cutoff k =7, window =0.5 s	One-way ANOVA	Thresholding	All channels
			Time–Frequency	wICA	Wavelet coefficients	n.r.			
			Time/Space	ICA with infomax (ADJUST)	Kurtosis, Spatial Average/Variance Difference, max Epoch Variance, Spatial Eye Difference, Generic Discontinuities Spatial Feature	n.r.			
Inoue et al. (2019) [59]	**R**: BPF (0.5–60 Hz)	5.12 s, 2.56 s; overlap n.r.	Frequency	FFT	Mean power spectrum	FFT length = 1024 points, window function = Hanning, frequency resolution = 0.2 Hz	n.c.	Thresholding	Multi-channel
Blum et al. (2019) [85]	**R**: FIR LPF (40 Hz), FIR HPF (0.25 Hz)	0.5 s; overlap n.r.	Time	rASR (PGA)	Mean and standard deviation	flatline = 1, hp = (0.25, 0.95) Hz, channel = 0.9, noisy = 3, burst = 2, window = 0.3 s, cutoff k = 1, stepsize = 16, maxdims = 1	n.c.	Thresholding	Multi-channel
Butkevičiūtė et al. (2019) [86]	n.c.	n.r.	Time	EMD	IMFs	n.r.	n.c.	Thresholding	n.r.
Albuquerque et al. (2019) [87]	**R**: Downsampling (250 Hz), BPF (0.5–45 Hz)	4 s; overlap n.c.	Time–frequency	wICA	Wavelet coefficients	n.r.	One-way ANOVA; mRMR	Thresholding	All channels
Liu et al. (2019) [88]	**SS**: BPF (20–100 Hz) (EMG); n.r. (EEG)	10 s; overlap n.r.	Time	FMEMD and CCA	Autocorrelation	n.r.	N-channel (N = 3–8) random selection	Thresholding	All channels
Casadei et al. (2020) [89]	**R**: digital BPF (8–14 Hz)	0.09 s, 0.1 s; overlap n.r.	Time	Band-pass filter	Amplitude and phase	filter order = 150, time window = 0.3 s	n.c.	Thresholding	Single-channel
Islam et al. (2020) [90]	**R/SS**: BPF (0.5–64 Hz), Notch (50 Hz)	n.r.	Time	Infomax ICA	ICs (Max MI)	No. of ICs = No. of channels	n.c.	Topographic maps, spectrum, autocorrelation analysis	All channels
Noorbasha et al. (2020) [98]	n.r.	8 s; overlap n.c.	Time	SVD	Covariance matrix eigenvalues	n.r.	n.r.	IMDL	Single-channel
Dey et al. (2020) [92]	**R**: Notch (60 Hz)	128 s; 80% overlap	Time	Windowing	Time series	128 s, 80% overlap	Correlation; *t*-test	MLP	All channels
Liu et al. (2021) [93]	**R**: BPF (5-65 Hz), Notch (50 Hz)	n.r.	Time	RLS Adaptive Filtering	EMG-related coefficients	optimization method = RLS, reference signal = EMG, length = n.r.		Weighted least squares cost function minimization	Single-channel + EMG reference
Kumaravel et al. (2021) [94]	**R**: LPF (40 Hz), HPF (0.15–0.3 Hz)	10 s; overlap 50–75%	Time	ASR (PCA)	Energy of components	cutoff k > 8, (other parameters n.r.)	n.c.	Thresholding	All channels
Shahbakhti et al. (2021) [95]	n.r.	3 s; overlap n.r.	Frequency	VME-DWT	DWT skewness	modes = 12, compactness coefficient = 3000, threshold value = 0.1	n.c.	Thresholding	Single-channel
Sha’abani et al. (2021) [96]	**SS**: HPF (0.5 Hz), Normalization	n.r.	Time	EEMD	IMFs correlation/amplitude	ensembles = 200, noise amplitude = 0.4, decomposition level = 3	n.c.	OD	Single-channel
Aung et al. (2021) [97]	n.c.	n.r.	Time	M-mDistEn	Entropy measures	time delay = 1, dimension for the reconstruction of the phase space = 3, r=0.2× SD, n=2, bins = 64, fuzzy width = 0.3, fuzzy step = 2, scales = 1–15	n.c.	QDA	Single-channel
Zhang et al. (2021) [58]	n.a.	n.r.	Time–frequency	DWT	Wavelet coefficients (edge)	mother wavelet = Haar, decomposition levels = 6	n.c.	Thresholding	Single-channel
			Frequency	FFT	Kurtosis	step size = 1			
					MAD	n.r.			
Noorbasha et al. (2021) [98]	**R**: BPF (0.5–30 Hz)	n.r.	Time	SVD	Eigenvectors local mobility	n.r.	n.c.	SSA	Single-channel
Ingolfsson et al. (2022) [99]	n.c.	1 s; overlap n.r.	Frequency	FFT	Energy	n.r.	TPOT	MMC	Single-channel
			Time–frequency	DWT	Energy	mother wavelet = Haar, decomposition levels = n.r.			
Chen et al. (2022) [100]	**R1/SS**: HPF (0.5 Hz); **R2**: HPF (0.5 Hz), Re-sampling (250 Hz), Re-ref., z-score	2 s; overlap n.r.	Time	MEMD + CCA	Autocorrelation, FD, skewness, kurtosis	MEMD: directions number = 16, noise channels 3; CCA: n.r.;	n.c.	OD based on one-class SVM	**R1/SS, R2** (ii): All channels; **R2** (i): multi-channel
			Frequency		Total power, peak frequency				
Occhipinti et al. (2022) [101]	**R**: BPF (1–40 Hz), Notch (50 Hz), detrending, normalization	n.r.	Time–frequency	NA-MEMD	IMFs	No. of IMFs = n.r., noise channels = 6, reference signal = 2 microphones and 1 accelerometer	n.c.	Thresholding	Multi-channel
Paissan et al. (2022) [102]	**SS**: BPF (1–80 Hz), Notch (50 Hz) (EEG); BPF (0.3–10 Hz) (EOG); BPF (1–120 Hz), Notch (50 Hz) (EMG)	n.r.	Time	1-D Convolutional layers	Convolutional feature maps	epochs = 100, optimizer = Adam, kernel length = 3	n.c.	Linear layers + softmax function (argmax)	Single-channel
Peh et al. (2022) [104]	**R**: 4th order Butterworth, Notch (60 Hz), 4th order HPF (1 Hz), Downsampling (128 Hz)	0.5 s; 25% overlap	Time	Transformer- enhanced CNN with BM loss	Statistical + correlation features	input length = 0.5 s, overlap = 25%, kernel length = 3, optimizer: Adam, learning rate = $10^{-4}$, batch size = 1000	n.r.	CatBoost classifier	Single-/ multi-channel
Brophy et al. (2022) [105]	n.c.	4 s, 2 s; overlap n.r.	Time	CNN	Convolutional feature maps	input size = 640 (PLN), 512 (Ocular), 1024 (Muscular), other parameters = n.r.	n.c.	Fully connected layer + sigmoid activation (thresholding)	Single-channel
Xiao et al. (2022) [106]	**R**: BPF (1–40 Hz)	n.r.	Frequency	ASR (PCA)	Energy of frequency components	n.r.	n.r.	Thresholding	All channels
Arpaia et al. (2022) [107]	**R**: Baseline-based normalization, BPF (1–40 Hz)	n.r.	Time	ASR (PCA)	Time series component	cutoff k = 25 (non-aggressive)	10 times channels random selection	Thresholding	Multi-channel
Noorbasha et al. (2022) [108]	n.r.	5 s; overlap n.c.	Time–Frequency	SWT	Multiple frequency bands coefficients	decomposition levels = 6	n.c.	GSTV filter	Single-channel
Zhang et al. (2022) [109]	**SS**: Resampling (250 Hz), Notch, Standardization	4.1 s; overlap n.r.	Encoded pattern feature domain	MARSC	Encoded feature map	input size = 1025, signal slice length = 20, overlapped samples = 5, signal segments = 5, batch size = 5, optimizer = Adam, learning rate = 0.001	n.c.	GRU (discriminator and encoder)	All channels
Jayas et al. (2023) [110]	**R**: HPF (0.5 Hz), Notch (50/100 Hz), FIR BPF bands, z-score normalization	1 s, 2 s, 5 s; overlap n.c.	Time	n.r.	Amplitude RMS; amplitude Kurtosis; amplitude skewness; SNR; ACF; ZCR; Maximum Gradient	n.r.	n.c.	Random Forest	n.r.
			Frequency	n.r.	Power mean; Spectral entropy	n.r.			
Narmada et al. (2023) [111]	n.c.	n.r.	Time–frequency	EMD + DWT	Wavelet coefficients	n.r.	n.c.	I-CycleGAN + OS-EHO thresholding	n.r.
Mahmud et al. (2023) [112]	**R**: Resampling, Baseline drift correction, z-score and range normalization, Notch (50 Hz)	4 s, 2 s; overlap n.r.	n.r.	Multi-resolution spatial pooling	Convolutional feature maps	weight regulator value = 0.5, kernel size = 3, other parameters n.r.	n.c.	UNet-style encoder-decoder structure + deep supervision	Single-channel
Jiang et al. (2023) [113]	**R/SS**: Real time BPF (3–100 Hz), SSA LPF on eyeblink	1.5 s; overlap n.r.	Frequency	VME	Mode function	n.r.	n.c.	Thresholding	Single-channel
Cui et al. (2023) [60]	**SS**: BPF (1–80 Hz), Notch (powerline), Resampling (256 Hz) (EEG); BPF (0.3–10 Hz) (EOG); BPF 1–120 Hz (EMG), etc.	2 s; overlap n.r.	n.r.	Convolutional layers + BiGRU	Convolutional feature maps	kernel size = 9, convolutional layers channels = 32, optimizer = Adam, learning rate = 5 × $10^{-5}$	n.c.	FC layer + sigmoid activation (thresholding)	Single-channel
Kaongoen et al. (2023) [61]	**SS**: Downsampling (256 Hz), Notch (50 Hz), BPF (1–50 Hz)	3 s; no overlap	Time	ASR	Mean, SD	ASR: cutoff k = 4 and 5, window length = 500 ms	n.c.	Thresholding	Single-channel
			Time–frequency	WT		decomposition levels = 4			
Kumaravel et al. (2023) [115]	**R1**: LPF (40 Hz), HPF (0.15–0.3 Hz)	1 s, 10 s; overlap n.r.	Time	ASR (PCA)	Mean, SD	IMU-based calibration, other parameters n.r.	n.c.	Thresholding	Multi-channel
	**R2**: HPF (1 Hz)	1 s; overlap n.c.							
Li et al. (2023) [116]	**SS**: BPF (0.5–40 Hz), Notch (50 Hz) (EEG/EOG); **R**: BPF (1–80 Hz) + Notch (50 Hz) (EEG/EMG)	5 s; overlap n.r.	Frequency	ResUnet1D	Convolutional feature maps (1D-CNN)	kernel size = 1 × 3, 1 × 5, 1 × 7, optimizer = Adam, learning rate = 5 × 10^−5^, batch size = 256	n.c.	RNN	Single-channel
Yin et al. (2023) [118]	n.r.	n.r.	Time/Space	CNN	Convolutional feature maps	Learning rate = 1 × 10^−4^, batch size = 128, optimizer = Adam, kernel size = 3, activation function = Softmax	n.c.	GCTNet discriminator	Multi-channel
Chen et al. (2023) [120]	n.r.	2 s.	n.r.	Tranformer	Attentional maps	Learning rate = 3 × 10^−4^, batch-size = 50, optimizer = AdamW, kernel size = 1	n.c.	Denoiseformer Transformer- based decoder	Single-channel
Hermans et al. (2023) [121]	Band-pass (0.27–30 Hz), Notch (50 Hz)	1 s.	n.r.	CNN	CNN autoencoder	Learning rate = 1 × 10^−3^, batch size = 87, optimizer = Adam, kernel size = 1, activation function = ReLU	n.c.	CNN classifier	Multi-channel
O’Sullivan et al. (2023) [119]	n.r.	n.r.	n.r.	Convolutional Kernel Transform, CNN	n.r.	Layers No. = 3, Filters No. = 32, Kernel Size = 3, stride length = 1, Pooling Size = 8, pooling stride length = 4, activation function = ReLU, learning rate = 0.0009, optimiser = Adam	n.c.	CNN binary probability outputs	Multi-Channel
Xing et al. (2024) [132]	**SS**: Detrend, downsampling (200 Hz), BPF (1–50 Hz), HPF (1 Hz), Normalization. **R**: n.c.	2 s; overlap n.c.	Time	1-D convolution layers	Convolutional feature maps	optimizer = Adam, learning rate = 0.001, epochs = 1000, input segments = 4	n.c.	n.c. (clean signal reconstruction)	Single-channel
Bahadur et al. (2024) [122]	**SS**: BPF (0.5–40 Hz)	n.r.	Time–frequency	DWT	Wavelet coefficients	mother wavelet = Haar, decomposition levels chosen from $f_{s}$ to cover 0.1–16 Hz ocular band	n.c.	Thresholding	All channels
Arpaia et al. (2024) [123]	**R**: Notch (50 Hz), HPF (1 Hz)	0.5 s; overlap n.c.	Time	MEMD + ASR (PCA)	IMFs	ASR: window length = 0.5 s, cutoff k = 9; MEMD: n.r.;	n.c.	Thresholding	Multi-channel
Ingolfsson et al. (2024) [124]	n.c.	1 s, 2 s, 4 s, 8 s; overlap n.r.	Time–frequency	DWT	Energy features	mother wavelet = Haar; decomposition levels = 4	TPOT	TPOT (train) + MCCP (embedded)	All channels
			Frequency	FFT	High-frequency energy	n.r.			
Saleh et al. (2024) [125]	n.r.	4 s; overlap n.c.	Time	1-D convolution layers	Convolutional feature maps	n.r.	n.c.	n.c. (clean signal reconstruction)	Single-channel
Nair et al. (2025) [126]	n.c.	n.r.	Time/Space	CNN	Convolutional feature maps	n.r.	n.c.	FC layer + sigmoid activation (thresholding)	Multi-channel

## Data Availability

The data presented in this study are available in the research engines presented in Section 2.

## Supplementary Materials

The following supporting information can be downloaded at: https://www.mdpi.com/article/10.3390/s25185770/s1, Table S1: PRISMA-S Checklist.

## Abbreviations

The following abbreviations are used in this manuscript:

**ACF**	Autocorrelation Function
**ADJUST**	Artefact Detector based on the Joint Use of Spatial and Temporal features
**AMRC**	Amplitude Modulation Rate of Change
**ANC**	Adaptive Noise Canceler
**ANFIS**	Adaptive Noise Cancellation System
**ANOVA**	Analysis of Variance
**APF**	Adaptive Predictor Filter
**ASR**	Artifact Subspace Reconstruction
**AUC**	Area Under the Curve
**BC**	Binary Classification
**BiGRU**	Bidirectional Gated Recurrent Unit
**BM**	Belief Matching
**BPF**	Band-Pass Filter
**CC**	Correlation of Coefficient
**CCA**	Canonical Correlation Analysis
**CCR**	Correct Classification Rate
**CNN**	Convolutional Neural Network
**CSED**	Cumulative Sum of Squared Error Difference
**CSMC**	Corrugator Supercilii Muscle Contraction
**DWT**	Discrete Wavelet Transform
**DSNR**	Difference in Signal-to-Noise Ratio
**DTW**	Dynamic Time Warping
**EAWICA**	Enhanced Automatic Wavelet ICA
**EC**	Eyes Closed
**ECG**	Electrocardiogram
**EEG**	Electroencephalogram
**EEMD**	Ensemble Empirical Mode Decomposition
**EII**	Electric Impedance Imbalance
**EM**	Expectation–Maximization
**EMD**	Empirical Mode Decomposition
**EMG**	Electromyogram
**EMI**	Electromagnetic Interference
**EO**	Eyes Open
**EOG**	Electrooculogram
**ERP**	Event-Related Potential
**ETI**	Electrode-Tissue Impedance
**FC**	Fully Connected
**FCBF**	Fast Correlation-Based Filter
**FD**	Fractal Dimension
**FDR**	False Discovery Rate
**FIR**	Finite Impulse Response
**FMEMD**	Fast Multivariate Empirical Mode Decomposition
**FFT**	Fast Fourier Transform
**FORCe**	Wavelet + ICA (SOBI) + Thresholding
**FP-h**	False Positive rate per hour
**FPM**	False Positive rate per Minute
**FPR**	False Positive Rate
**FTR**	Frequency-Tagging Response
**GAN**	Generative Adversarial Network
**GRU**	Gated Recurrent Unit
**GRU-MARSC**	Gated Recurrent Unit-based Multi-type Artifact Removal algorithm for Single-Channel
**GSTV**	Group Sparsity Total Variation
**GWO**	Gray Wolf Optimization
**HPF**	High-Pass Filter
**HPO**	HyperParameter Optimization
**ICA**	Independent Component Analysis
**ICA-W**	Independent Component Analysis–Wavelet
**ICs**	Independent Components
**I-CycleGAN**	Improved CycleGAN
**IMDL**	Integrated Minimum Description Length
**IMFs**	Intrinsic Mode Functions
**IMU**	Inertial Measurement Unit
**ISD**	Index of Spectral Deformation
**KNN**	k-Nearest Neighbors
**LDA**	Linear Discriminant Analysis
**LiMC**	Limb Muscle Contraction
**LaMC**	Laryngeal Muscle Contraction
**LLMS**	Leaky Least Mean Squares
**LMM**	Local Maximal and Minimal
**LMS**	Least Mean Square
**LogP**	Log Power
**LPF**	Low-Pass Filter
**LZC**	Lempel–Ziv Complexity
**MAD**	Median Absolute Deviation
**MAE**	Mean Absolute Error
**MCA**	Morphological Component Analysis
**MCAF**	Multi-Channel Adaptive Filtering
**MARSC**	Multi-type Artifact Removal algorithm for Single-Channel
**MC**	Multi-class Classification
**MCC**	Matthews Correlation Coefficient
**MCCP**	Minimal Cost-Complexity Pruning
**MEMD**	Multivariate Empirical Mode Decomposition
**MFE**	Morphological Feature Extraction
**MI**	Mutual Information
**MMC**	Multi-class Multi-output Classification
**MaMC**	Masseter Muscle Contraction
**MeMC**	Mentalis Muscle Contraction
**MODWT**	Maximal Overlap Discrete Wavelet Transform
**MRA**	MultiResolution Analysis
**MSC**	Magnitude Square Coherence
**MSDW**	Multi-window Summation of Derivatives within a Window
**MSE**	Mean Square Error
**MV-EMD**	Multivariate EMD with CCA
**M-mDistEn**	Multiscale Modified-Distribution Entropy
**MLP**	Multilayer Perceptron
**mRMR**	Minimum Redundancy Maximum Relevance
**NA-MEMD**	Noise-Assisted Multivariate Empirical Mode Decomposition
**NMC**	Nasalis Muscle Contration
**NLMS**	Normalized Least Mean Square
**NSR**	Noise-to-Signal Ratio
**OD**	Outlier Detection
**OOcMC**	Orbicularis Oculi Muscle Contraction
**OOrMC**	Orbicularis Oris Muscle Contraction
**OS-EHO**	Opposition Searched–Elephant Herding Optimization
**PCA**	Principal Component Analysis
**PCMC**	Posterior Cervical Muscle Contraction
**PLN**	Power-Line Noise
**PMC**	Pharyngeal Muscle Contraction
**PGA**	Principal Geodesic Analysis
**PSD**	Power Spectral Density
**PSNR**	Peak Signal-to-Noise Ratio
**PPV**	Positive Predictive Value
**QDA**	Quadratic Discriminant Analysis
**RLS**	Recursive Least-Squares Adaptive
**RMS**	Root Mean Square
**RMSE**	Root Mean Squared Error
**RCs**	Reconstructed Components
**ResCNN**	Residual Convolutional Neural Network
**ResUnet1D**	1D Residual U-Net Semantic Segmentation Network
**RNN**	Recurrent Neural Network
**RP**	Relative Power
**RRMSE**	Relative Root Mean Squared Error
**RRMSEf**	Relative Root Mean Squared Error in time domain
**RRMSEt**	Relative Root Mean Squared Error in frequency domain
**RSD**	Relative Spectral Difference
**SAR**	Signal-to-Artifact Ratio
**SBF**	Stop-Band Filter
**SD**	Standard Deviation
**SDW**	Summation of Derivatives within a Window
**ShMC**	Shoulder Muscle Contraction
**SSA**	Singular Spectrum Analysis
**SubMc**	Submentalis Muscle Contraction
**SE**	Shannon Entropy
**SEF**	Spectral Edge Frequency
**SG**	Savitzky–Golay Filter
**SOBI**	Second Order Blind Identification
**SNR**	Signal-to-Noise Ratio
**SVM**	Support Vector Machine
**SSVEP**	Steady-State Visual Evoked Potentials
**SWT**	Stationary Wavelet Transform
**STFT**	Short-Time Fourier Transform
**SVD**	Singular Value Decomposition
**TEN**	Thermal Electronics Noise
**TeMC**	Temporalis Muscle Contraction
**ToMC**	Tongue Muscle Contraction
**TFA**	Time–Frequency Analysis
**TPR**	True Positive Rate
**TPOT**	Tree-based Pipeline Optimization Tool
**vEOG**	vertical Electrooculogram
**VME**	Variational Mode Extraction
**VME-DWT**	Variational Mode Extraction with Discrete Wavelet Transform
**WICs**	Wavelet Independent Components
**wICA**	Wavelet-enhanced Independent Component Analysis
**WT**	Wavelet Transform
**ZCR**	Zero Crossing Rate
**ZMC**	Zygomaticus Muscle Contraction
